# Comprehensive Profiling of Phytocannabinoids and Semisynthetic Cannabinoids in Seized Materials from Northern Italy

**DOI:** 10.1021/acsomega.6c00941

**Published:** 2026-05-27

**Authors:** Sara Casati, Roberta F. Bergamaschi, Lea Sicuro, Alessandro Ravelli, Sofia Vanerio, Valentina Orsi, Camilla Ronco, Enrica Torretta, Alberto Rimoldi, Gabriella Roda, Alessio Battistini, Luca Mollica, Paola Rota

**Affiliations:** † Laboratorio di Tossicologia Forense, Dipartimento di Scienze Biomediche, Chirurgiche e Odontoiatriche, 9304 Università degli Studi di Milano, via Luigi Mangiagalli 37, 20133 Milan, Italy; ‡ 189821 Fondazione IRCCS Ca’ Granda Ospedale Maggiore Policlinico, via Francesco Sforza 35, Milan 20135, Italy; § Dipartimento di Biotecnologie Mediche e Medicina Traslazionale, 70301 Università degli Studi di Milano, via Fratelli Cervi 93, Segrate 20054, Italy; ∥ Dipartimento di Scienze Farmaceutiche, 9304 Università degli Studi di Milano, via Trentacoste 2, Milan 20134 Italy; ⊥ Dipartimento di Scienze Biomediche, Chirurgiche e Odontoiatriche, 9304 Università degli Studi di Milano, via Della Commenda 10, Milan 20121, Italy; # Institute for Molecular and Translational Cardiology (IMTC), San Donato Milanese, Milan 20097, Italy

## Abstract

In recent years, semisynthetic cannabinoids (SSCs) have emerged in commercial and illicit markets as alternatives designed to circumvent regulations targeting Δ^9^-tetrahydrocannabinol (Δ^9^-THC). However, their pharmacological properties, potency, and toxicological profiles remain poorly characterized, raising concerns about potential risks to consumers. In the present study, a liquid chromatography–tandem mass spectrometry (LC-MS/MS) method was developed and validated for the identification and quantification of 31 phytocannabinoids and SSCs, including acetylated forms, in*Cannabis* *-*derived products. The initial identification of Δ^8^- and Δ^9^-tetrahydrocannabinol acetate (Δ^8^-THCOAc, Δ^9^-THCOAc), 9*R-* and 9*S*-hexahydrocannabinol acetate (9*R*,9*S*-HHCOAc), and cannabinol acetate (CBNOAc) was achieved using in-house synthesized reference materials. The LC separation successfully resolved multiple epimeric and isomeric cannabinoid structures, and the validated method was applied to 151*Cannabis*-derived products seized in Northern Italy between 2024 and 2025. Over the study period, a total of 93% of the seized*Cannabis* *-*derived products contained SSCs (>LOQ), including 49% of flowers and 97% of resins. Δ^9^-THCOAc was the most frequently identified SSC (85%), followed by Δ^9^-tetrahydrocannabiphorol (Δ^9^-THCP) (61%), Δ^8^-tetrahydrocannabinol (Δ^8^-THC) (18%), and Δ^8^-THCOAc (6%). In addition, molecular docking simulations were performed on cannabinoid receptors 1 and 2 (CB1 and CB2) to provide a structure-based framework for interpreting how acetylation and side-chain modification influence ligand–receptor interactions, indicating increased binding propensity for acetylated and side-chain-extended derivatives. SSCs represent an emerging class of cannabinoids with a rapidly increasing popularity and distinct receptor binding affinities. Despite their widespread availability, systematic investigations are urgently needed to assess their safety profiles and their potential public health implications.

## Graphical abstract

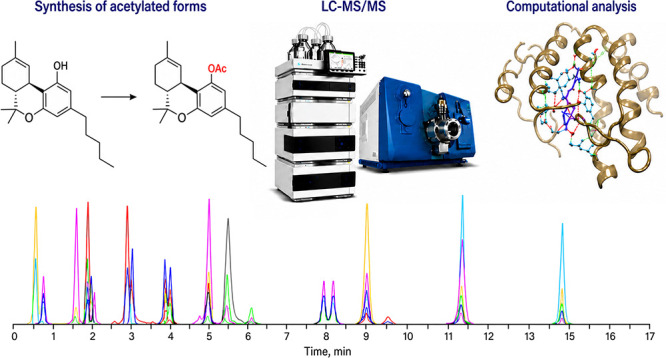

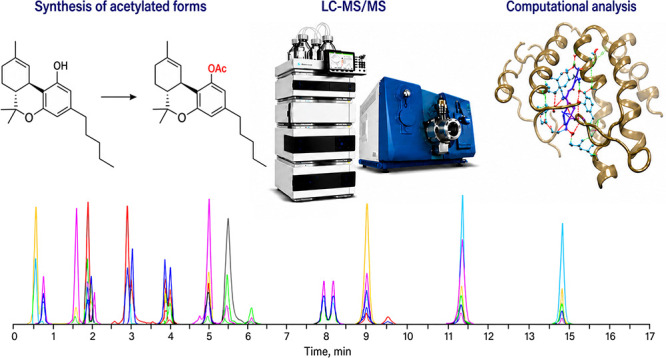

## 1 Introduction

*Cannabis sativa* L. produces a chemically diverse array of phytocannabinoids, with more than 150 compounds identified to date.^1^ The term *phytocannabinoids* refers to cannabinoids naturally occurring in*Cannabis sativa*, whereas *semisynthetic cannabinoids (SSCs)* denote compounds produced through chemical modification of these natural constituents (e.g., hydrogenation, acetylation, or structural derivatization). The major acidic phytocannabinoids, Δ^9^-tetrahydrocannabinolic acid (Δ^9^-THCA) and cannabidiolic acid (CBDA), undergo nonenzymatic decarboxylation during drying or heating, yielding Δ^9^-tetrahydrocannabinol (Δ^9^-THC) and cannabidiol (CBD), respectively (Figure 1). While Δ^9^-THC, the principal psychoactive constituent of*Cannabis*, largely defines the legal status of*Cannabis*-derived products, CBD is nonpsychoactive and has been extensively investigated for its potential therapeutic properties.^2^

**Figure 1:**
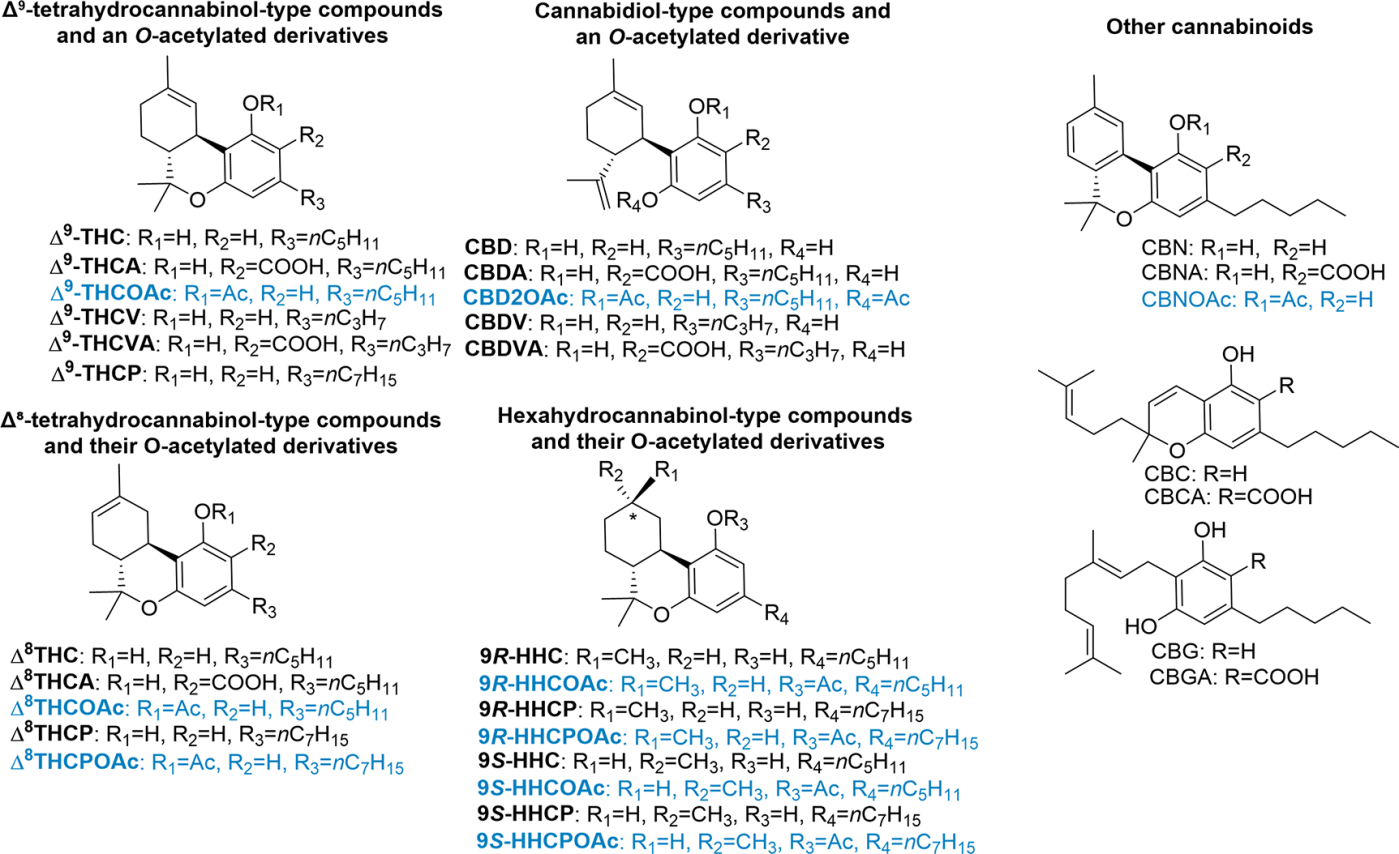
Structures of 31 phytocannabinoids and semisynthetic cannabinoids (acetylated forms in blue).

In recent years, CBD has also emerged as a key chemical precursor for the synthesis of SSCs. More generally, SSCs obtained through the chemical modification of CBD and/or Δ^9^-THC have increasingly been used as substitutes for Δ^9^-THC in commercial and illicit products, partly to circumvent regulatory controls focused on Δ^9^-THC.

Among these, Δ^8^-tetrahydrocannabinol (Δ^8^-THC) was one of the first compounds to gain widespread attention.^3^ Although Δ^8^-THC occurs naturally only in trace amounts in aged*Cannabis*,^4^ it can be readily produced from CBD via acid-catalyzed cyclization reactions, yielding mixtures of Δ^8^- and Δ^9^-THC.^5,6^

Since 2019, a rapidly expanding range of SSCs has been detected in treated herbal material, resins, vape liquids, and edibles.

HHC is most commonly encountered as a synthetic product,^7^ although trace amounts have been detected in*Cannabis* plants,^8–10^ where it is thought to arise as a degradation product of Δ^9^-THC,^9^ and it typically occurs as a mixture of 9*R*-HHC and 9*S*-HHC epimers,^7,11^ with epimeric ratios depending on the cannabinoid precursor used in its synthesis.

More recently, the heptyl homologues of HHC, collectively referred to as hexahydrocannabiphorol (HHCP), have emerged on the market.^12^ Structurally, HHCP shares features with both Δ^8^- or Δ^9^- THCP and HHC, combining an extended alkyl side chain with a hydrogenated cannabinoid scaffold.

A further emerging trend involves the marketing of acetylated derivatives of previously known cannabinoids (reported in blue in Figure 1). In particular, THCOAc analogues have been reported for Δ^8^- and Δ^9^-THC, as well as an acetylated derivative of CBD, commonly referred to as cannabidiol diacetate (CBD2OAc) and for HHC and HHCP, known as HHCOAc and HHCPOAc, respectively. These compounds are generally obtained via acetylation of the corresponding parent cannabinoids using acetic anhydride, acetyl chloride, or other conventional acetylating reagents.^13,14^

To the best of our knowledge, fully detailed and reproducible synthetic routes for HHCOAc and HHCPOAc have not been reported, although early literature describes related acetylation procedures without complete experimental details;^10^ however, these compounds are presumed to be prepared by acetylation of their corresponding nonacetylated precursors using approaches analogous to those employed for the preparation of THC acetates.

This rapid diversification of SSCs highlights the urgent need for selective analytical methods capable of distinguishing closely related isomers, epimers, and homologues in seized materials, as also evidenced by the recent works of Hundertmark et al.,^15^ Tanaka et al.,^14^ and Holt et al.^16^ Such structural modifications are pharmacologically relevant, as structure–activity relationship studies indicate that relatively small changes, including alkyl side-chain extension or acetylation, can markedly influence cannabinoid receptor interactions.^7,17,18^ Cannabinoid receptor type 1 (CB1) is the primary receptor responsible for the psychoactive effects of THC and cannabinoid receptor type 2 (CB2) is associated with an immunomodulatory role.^19^ In particular, extension of the alkyl side chain generally increases CB1 receptor potency,^19–21^ while the 9*R* epimers of hydrogenated cannabinoids exhibit higher affinity and cannabimimetic activity than their 9*S* counterparts.^7^ Despite anecdotal early reports on certain acetylated cannabinoids,^22^ reliable contemporary pharmacological and toxicological data for many SSCs remain scarce.

In response to these challenges, in the present study a liquid chromatography–tandem mass spectrometry (LC-MS/MS) method was developed and validated for the identification and quantification of 31 SSCs and major phytocannabinoids in*Cannabis* *-*derived products. The initial identification of Δ^8^-THCOAc, Δ^9^-THCOAc, 9*R,S*-HHCOAc, and cannabinol acetate (CBNOAc) was performed by using in-house synthesized reference material. The validated method was successfully applied to 151*Cannabis*-derived products seized in Northern Italy between 2024 and 2025. In addition, molecular docking simulations were performed on cannabinoid receptors CB1 and CB2 to provide a structure-based framework for interpreting how acetylation and side-chain modification influence ligand–receptor interactions. Docking results support an increased CB1 binding propensity for acetylated and side-chain-extended derivatives in agreement with the previously documented pharmacological activity of the investigated compounds.

## 2 Materials and Methods

### 2.1 Chemicals and Reagents

Ultrapure water (H_2_O), acetonitrile (ACN), ethanol (EtOH), and methanol (MeOH) were of analytical grade and purchased from Carlo Erba (Milan, Italy). Formic acid (98%–100%) was purchased from Merck (Darmstadt, Germany). Δ^8^-THCOAc, Δ^9^-THCOAc, 9*R*- and 9*S*-HHCOAc, and CBNOAc were initially custom-synthesized by our laboratory as described in paragraph 2.2. The reference materials cannabichromene (CBC) (1 mg/mL in MeOH), cannabidivarin (CBDV) (1 mg/mL in MeOH), cannabigerol (CBG) (1 mg/mL in MeOH), cannabinol (CBN) (1 mg/mL in MeOH), Δ^8^-THC (1 mg/mL in MeOH), Δ^8^-THCP (1 mg/mL in MeOH), and Δ^9^-THC (1 mg/mL in MeOH) were purchased from Merck (Darmstadt, Germany). CBD (10 mg/mL in MeOH) was purchased from PhytoLab GmbH & Co. KG (Vestenbergsgreuth, Germany). CBD2OAc (5 mg/mL in ACN), Δ^9^-THCV (1 mg/mL in MeOH), Δ^8^-THCOAc (1 mg/mL in ACN), Δ^8^-THCA (1 mg/mL in ACN), Δ^8^-Tetrahydrocannabiphorol acetate (Δ^8^-THCPOAc) (10 mg/mL in ACN), CBNOAc (1 mg/mL in ACN), Δ^9^-THCP (1 mg/mL in ACN) were purchased from Cayman (Ann Arbor, Michigan, USA). Cannabinoids Acids 7 Standard (cannabichromenic acid (CBCA), CBDA, cannabidivarinic acid (CBDVA), cannabigerolic acid (CBGA), cannabinolic acid (CBNA), Δ^9^-THCA, and Δ^9^-tetrahydrocannabivarinic acid (Δ^9^-THCVA) at the concentration of 1 mg/mL in ACN) was purchased from Restek (Bellefonte, Pennsylvania, USA). 9*R*-HHC (0.1 mg/mL in MeOH), 9*R*-HHCOAc (0.1 mg/mL in MeOH), 9*R*-HHCP (0.1 mg/mL in MeOH), 9*R*-HHCPOAc (0.1 mg/mL in MeOH), 9*S*-HHC (0.1 mg/mL in MeOH), 9*S*-HHCOAc (0.1 mg/mL in MeOH), 9*S*-HHCP (0.1 mg/mL in MeOH), and 9*S*-HHCPOAc (0.1 mg/mL in MeOH) were obtained from Comedical (Trento, Italy).

The internal standards (IS) CBD-*d*_3_ (0.1 mg/mL in MeOH), Δ^9^-THC-*d*_3_ (0.1 mg/mL in MeOH), and Δ^9^-THCA-*d*_3_ (0.1 mg/mL in ACN) were purchased from Merck (Darmstadt, Germany).

### 2.2 Synthesis of Cannabinoid Acetates

#### 2.2.1 Chemistry

All chemicals and solvents used were of analytical grade and purchased from Sigma-Aldrich (St. Louis, MO). Solvents were dried using standard methods and distilled before use. The progress of all reactions was monitored by thin-layer chromatography (TLC) carried out on 0.25 mm Sigma-Aldrich silica gel plates (60 F254) using UV light, anisaldehyde/H_2_SO_4_/EtOH solution. Flash chromatography was performed with normal-phase silica gel (Sigma-Aldrich 230–400 mesh silica gel). Nuclear magnetic resonance spectra were recorded at 298 K on a Bruker AM-500 spectrometer equipped with a 5 mm inverse-geometry broadband probe and operating at 500.13 MHz for ^1^H and 125.76 MHz for ^13^C. The ^1^H and ^13^C resonances were assigned by ^1^H–^1^H (COSY) and ^1^H–^13^C (HSQC and HMBC) correlation of two-dimensional (2D) experiments. Chemical shifts are reported in parts per million and are referenced for ^1^H spectra to a solvent residue proton signal (δ = 7.26 ppm for CDCl_3_) and for ^13^C spectra, to the solvent carbon signal (central line at δ = 77.00 ppm, for CDCl_3_). The ^1^H NMR data are tabulated in the following order: multiplicity (s = singlet, d = doublet, t = triplet, br = broad, m = multiplet, app = apparent), coupling constants are given in Hz, number of protons, and assignment of proton(s). In this context, the symbols α and β refer exclusively to substituents resonating at higher and lower chemical shift values (ppm), respectively, and do not indicate stereochemical configuration.

#### 2.2.2 General Acetylation Procedure

Starting from 1 mg of pure or purified starting material, the sample was diluted in CH_2_Cl_2_ (0.2 mL) and then treated with Et_3_N (0.05 mL) and Ac_2_O (0.025 mL). The reaction was stirred at 50 °C and monitored by TLC (Hexane/AcOEt 9:1, v:v). After 1 h, the reaction was quenched by the addition of MeOH (0.2 mL) and the solvent was removed under vacuum. The crude product was purified by flash chromatography (Hexane/AcOEt 95:5, v:v).

#### 2.2.3 Acetylation of Diastereomeric Mixture of 9*R*-HHC and 9*S*-HHC

^1^H-NMR assignments for the diastereomeric mixture of 9*R*-HHC and 9*S*-HHC acetates refer to the structure numbers in Figure 2:

**Figure 2:**
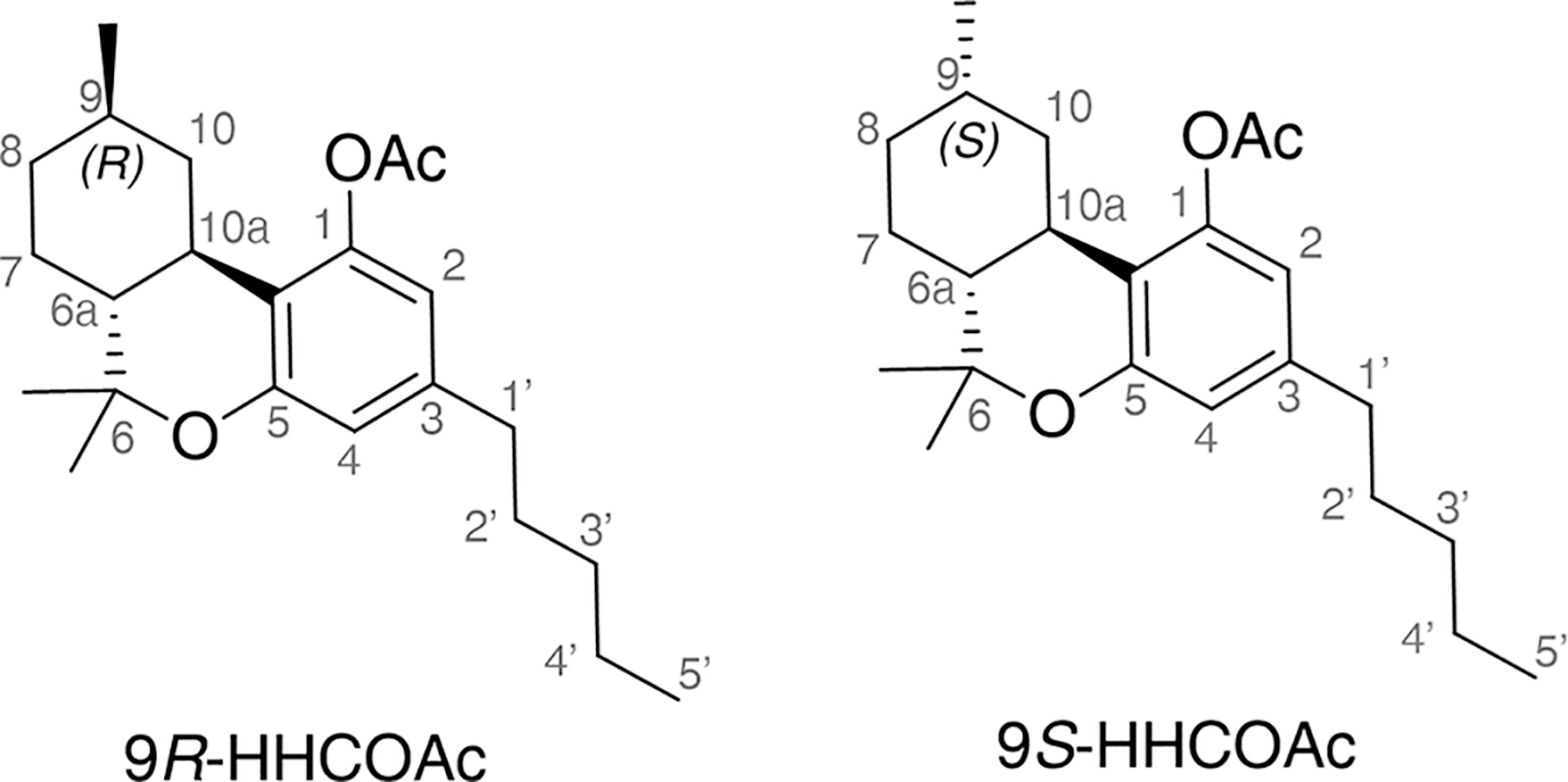
Structural formulas and atom numbering of *9R*- and *9S*-hexahydrocannabinol acetates.

Starting from 1 mg of a purified diastereomeric mixture (7:3 ratio, *9R:9S*) of 9*R*-HHC and 9*S*-HHC, and applying the general acetylation procedure, the desired acetylated mixture of 9*R*-HHCOAc and 9*S*-HHCOAc was achieved in high yield (1 mg, 89% and 7:3 diastereomeric ratio). ^1^H NMR analysis of the 9*R-*HHCOAc and 9*S-*HHCOAc mixture showed: ^1^H NMR (500 MHz, CDCl_3_) δ 6.53–6.51 (br s app, overlapping, H-4), 6.37–6.35 (br s app, overlapping, H-2), 2.60–2.29 (overlapping, Hα-10, H-10a, and 2H at C1′), 2.28 and 2.27 (2 × OCOCH_3_), 1.88–0.98 (overlapping, 2H at C7, 2H at C8, H-9, 2H at C2′, 2H at C3′, 2H at C4′, Hβ-10, and CH_3_ at C9), 1.45 (overlapping, 6a), 1.36 (overlapping, CH_3_α at C6), 1.06 (overlapping, CH_3_β at C6), 0.94 (d, *J* = 6.6 Hz, CH_3_ at C9), 0.88 (overlapping, t app, *J* = 6.7 Hz, H-5′), 0.80 (q app, *J* = 12.9 Hz, Hβ-10).

#### 2.2.4 Acetylation of Δ^8^-THC

^1^H and ^13^C assignments for Δ^8^-THCOAc refer to the structure numbers in Figure 3:

**Figure 3:**
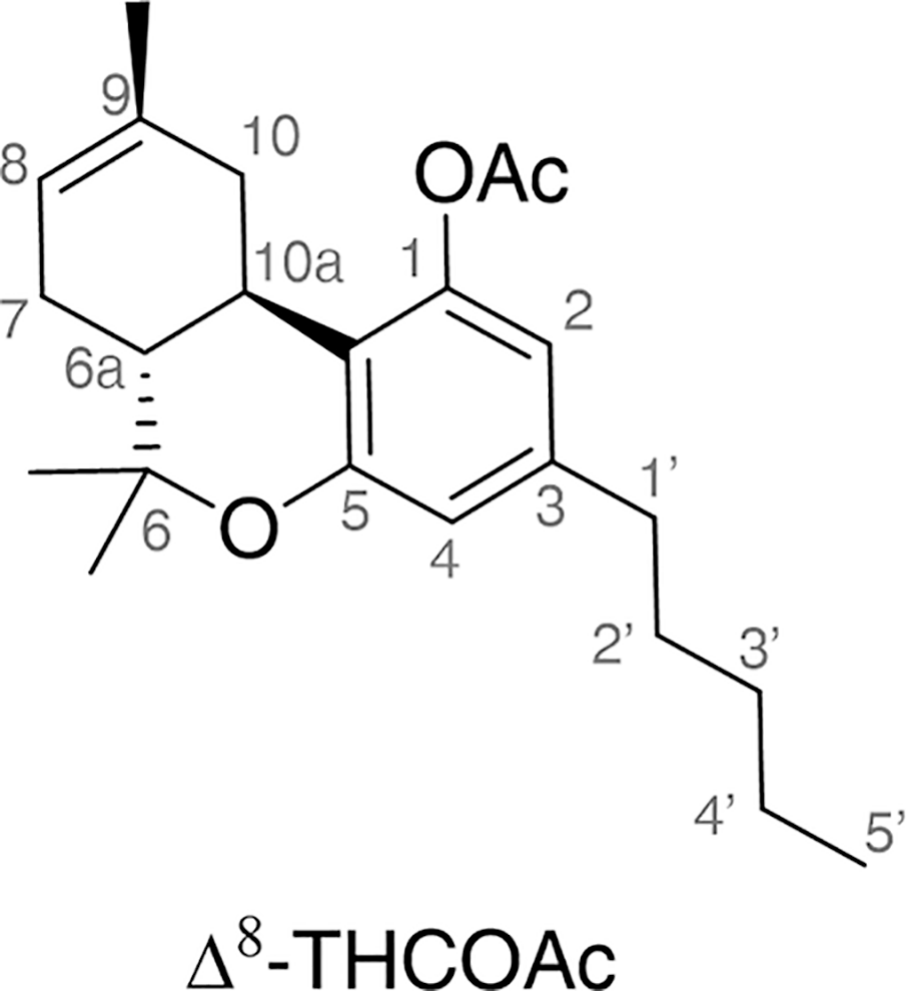
Structural formula and atom numbering of Δ^8^-THC acetate.

Starting from 1 mg of Δ^8^-THC and applying the general acetylation procedure, the desired Δ^8^-THCOAc was achieved in high yield (1 mg, 88%). ^1^H NMR and ^13^C NMR analysis of Δ^8^-THCOAc showed: ^1^H NMR (500 MHz, CDCl_3_) δ 6.56 (br d, *J* = 1.4 Hz, 1H, H-4), 6.40 (br d, *J* = 1.4 Hz, 1H, H-2), 5.42 (s app, 1H, H-8), 2.72 (dd, 1H, *J* = 4.4, *J* = 17.1 Hz, Hα-10), 2.59 (ddd, *J* = 4.4, *J* = 11.3, *J* = 12.9 Hz, 1H, H-10a), 2.50 (m, 2H, 2H at C1′), 2.28 (s, 3H, OCOCH_3_), 2.12 (m, 1H, Hα-7), 1.91 (m, 1H, Hβ-10), 1.84–1.72 (overlapping, 2H, Hβ-7, and H-6a), 1.68 (s, 3H, CH_3_ at C9), 1.62–1.50 (overlapping to water signal, 2H, 2H at C2′), 1.37 (s, 3H, CH_3_α at C6), 1.35–1.22 (overlapping, 4H, 2H at C3′ and 2H at C4′), 1.09 (s, 3H, CH_3_β at C6), 0.87 (t, *J* = 6.9 Hz, 3H, H-5′). ^13^C NMR (125 MHz, CDCl_3_) δ 169.0 (O*C*OCH_3_), 154.5 (C5), 142.8 (C1), 133.9 (C3), 122.7 (C9), 119.7 (C8), 116.0 (C11), 115.3 (C4), 114.3 (C2), 77.7 (overlapping to solvent signal, C6), 44.6 (C6a), 36.1 (C10), 35.3 (C1′), 31.7, 31.5 (C2′ and C3′), 30.5 (C10a), 27.7 (C7), 27.4 (CH_3_α at C6), 23.6 (CH_3_ at C9), 22.5 (C4′), 21.3 (OCO*C*H_3_), 18.5 (CH_3_β at C6), 14.0 (C5′).

#### 2.2.5 Acetylation of Δ^9^-THC

^1^H and ^13^C assignments for Δ^9^-THCOAc refer to the structure numbers in Figure 4:

**Figure 4:**
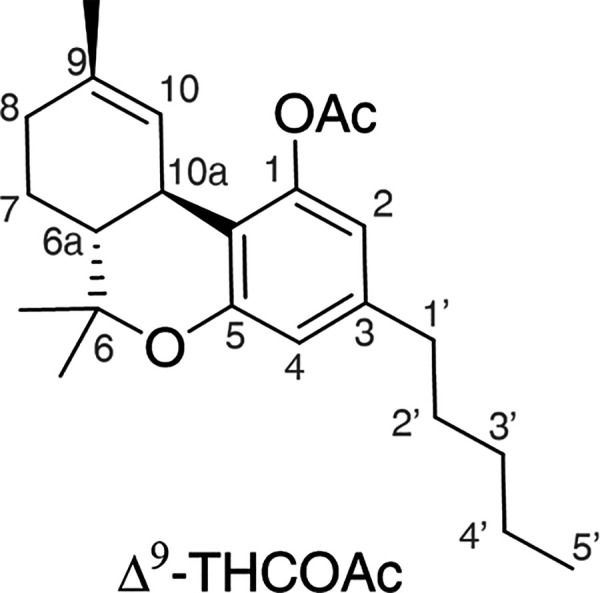
Structural formula and atom numbering of Δ^9^-THC acetate.

Starting from 1 mg of Δ^9^-THC and applying the general acetylation procedure, the desired acetylated Δ^9^-THCOAc was achieved in high yield (1 mg, 88%). ^1^H NMR and ^13^C NMR of Δ^9^-THCOAc analysis showed: ^1^H NMR (500 MHz, CDCl_3_) δ 6.54 (br d, *J* = 1.4 Hz, 1H, H-4), 6.4 (br d, *J* = 1.4 Hz, 1H, H-2), 5.97 (s app, 1H, H-10), 3.06 (br d, *J* = 10.1 Hz, 1H, H-10a), 2.52–2.45 (overlapping, 2H, 2H at C1′), 2.28 (s, 3H, OCOCH_3_), 2.16–2.09 (overlapping, 2H, 2H at C8), 1.89 (m, 1H, Hα at C7), 1.69–1.62 (overlapping, 4H, CH_3_ at C9 and H-6a), 1.61–1.52 (overlapping to water signal, 2H, 2H at C2′), 1.40 (s, 3H, CH_3_α at C6), 1.39–1.20 (overlapping, 5H, 2H at C3′, 2H at C4′ and Hβ at C7), 1.08 (s, 3H, CH_3_β at C6), 0.87 (t, *J* = 6.9 Hz, 3H, 3H at C5′).^13^C NMR (125 MHz, CDCl_3_) δ 168.9 (O*C*OCH_3_), 154.5 (C5), 149.3 (C1), 142.8 (C3), 134.9 (C9), 123.1 (C10), 115.3 (C4), 115.1 (C11), 114.0 (C2), 77.5 (overlapping to solvent signal C6), 45.6 (C6a), 35.4 (C1′), 34.1 (C10a), 31.5 (C3′), 31.1 (C8), 30.6 (C2′), 27.5 (CH_3_α at C6), 24.9 (C7), 23.5 (CH_3_ at C9), 22.6 (C4′), 21.3 (OCO*C*H_3_), 19.4 (CH_3_β at C6), 14.0 (C5′).

#### 2.2.6 Acetylation of CBN

^1^H and ^13^C assignments for CBNOAc refer to the structure numbers in Figure 5:

**Figure 5:**
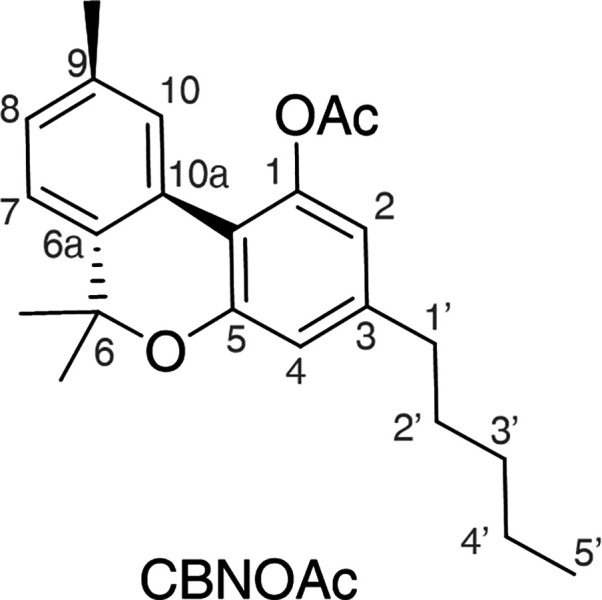
Structural formula and atom numbering of CBN acetate.

Starting from 1 mg of CBN and applying the general acetylation procedure, the desired CBNOAc was achieved in high yield (1 mg, 88%). ^1^H NMR and ^13^C NMR of CBNOAc: ^1^H NMR (500 MHz, CDCl_3_) δ 7.80 (s, 1H, H-10), 7.14 (br d, *J* = 7.9 Hz, 1H, H-7), 7.83 (br dd, *J* = 1.1 Hz, *J* = 7.9 Hz, 1H, H-8), 6.72 (br d, *J* = 1.7 Hz, 1H, H-4), 6.40 (br d, *J* = 1.7 Hz, 1H, H-2), 2.59–2.53, 2.36 (overlapping, 2H, 2H at C1′), 2.36 (s, 3H, CH_3_ at C9), 2.32 (s, 3H, OCOCH_3_), 1.66–1.57 (overlapping, 8H, 2H at C2′, CH_3_α at C6 and CH_3_β at C6), 1.37–1.28 (overlapping, 4H, 2H at C3′ and 2H at C4′), 0.89 (t, *J* = 6.9 Hz, 3H, H-5′). ^13^C NMR (125 MHz, CDCl_3_) δ 169.1 (O*C*OCH_3_), 154.3 (C5), 147.3 (C1), 144.5 (C3), 137.5 (C10a), 136.8 (C6a), 128.3 (C8), 126.8 (C9), 125.6 (C10), 122.8 (C7), 116.3 (C2), 115.8 (C4), 114.2 (C11), 77.7 (overlapping to solvent signal, C6), 35.5 (C1′), 31.4 (C3′), 30.4 (C2′), 29.7 (CH_3_α at C6), 26.9 (CH_3_β at C6), 22.5 (CH_2_ at C4′), 21.5 (CH_3_ at C9), 21.4 (CO*C*H_3_), 14.0 (C5′).

### 2.3 Standard Solutions, Calibrators, and Quality Control Samples

All the solutions were stored in the dark at −20 °C. Working solutions were prepared from stock solutions (0.1 or 0.01 mg/mL) by appropriate dilution in MeOH and used for the preparation of calibration curves and quality control (QC) samples at 1 μg/mL and 100 ng/mL for all compounds, and at 1 μg/mL for ISs (CBD-*d*_3_, Δ^9^-THC-*d*_3_, and Δ^9^-THCA-*d*_3_).

Calibration standards containing:

0, 0.5, 1, 2.5, 5, 10, 25 ng/mL (corresponding to 0, 1, 2.5, 5, 10, 25, 50% since 50 ng/mL was set as 100%) for Δ^8^-THC, Δ^9^-THC, Δ^8^-THCA, Δ^9^-THCA;
0, 1, 2.5, 5, 10, 25 ng/mL (corresponding to 0, 0.1, 0.25, 0.5, 1, 2.5% since 1 μg/mL was set as 100%) for CBC, CBCA, CBD, CBDA, CBDV, CBDVA, CBG, CBGA, CBN, CBNA, Δ^9^-THCV, Δ^9^-THCVA, Δ^9^-THCOAc, 9*R*- and 9*S*-HHC;
0, 1, 2.5, 5, 10, 25, 50 ng/mL (corresponding to 0, 0.001, 0.0025, 0.005, 0.01, 0.025, 0.05% since 100 μg/mL was set as 100%) for CBD2OAc, CBNOAc, Δ^8^-THCOAc, Δ^8^-THCP, Δ^8^-THCPOAc, Δ^9^-THCP, 9*R*-HHCOAc, 9*R*-HHCP, 9*R*-HHCPOAc, 9*S*-HHCOAc, 9*S*-HHCP, and 9*S*-HHCPOAc;
10 ng/mL for CBD-*d*_3_, Δ^9^-THC-*d*_3_, and Δ^9^-THCA-*d*_3_;

were freshly prepared for each analytical batch by adding suitable volumes of working solutions to 1 mL of MeOH.

### 2.4 Collection and Preparation of *Cannabis* and Cannabis-Derived Products Samples

An aliquot of 25 mg from each flower (*n* = 69), resin (*n* = 69), wax (*n* = 3), vape-liquid (*n* = 9), and powder (*n* = 1) sample was extracted with 2.5 mL of cyclohexane and mixed for 15 min at room temperature. 10 μL aliquot of the initial solution (S1) was added to 990 μL of MeOH to obtain a second diluted solution (S2). A third solution (S3) was prepared by diluting a 10 μL aliquot of the S2 in 990 μL of MeOH.

For LC-MS/MS analysis, each sample was prepared as follows:

– 10 μL aliquot of S2 was added to 10 μL of IS and 980 μL of MeOH (final dilution 1:10000);

– 50 μL aliquot of S3 was added to 10 μL of IS and 940 μL of MeOH (final dilution 1:200000);

– 5 μL aliquot of S3 was added to 10 μL of IS and 985 μL of MeOH (final dilution 1:2000000).

### 2.5 LC-MS/MS Analysis

Analyses were performed on a 1290 Infinity UHPLC system (Agilent Technologies, Palo Alto, CA, USA) coupled to a QTrap 5500 triple quadrupole linear ion trap mass spectrometer (Sciex, Darmstadt, Germany). Compounds were separated on a Kinetex HPLC XB-C18 column (100 mm × 2.1 mm i.d., 2.6 μm) (Phenomenex, CA, USA) using 0.1% formic acid in water (mobile phase A) and 0.1% formic acid in acetonitrile (mobile phase B).

The 17 min linear gradient was the following: from 0.00 to 4.80 min solvent B increased from 70% to 75% and held 75% from 4.80 to 7.00 min, then solvent B increased to 82% from 7.00 to 12.00 min, and to 90% from 12.00 to 12.10 min, then held at 90% to 15.00 min, decreased to 70% from 15.00 to 15.10 min, and finally held at 70% from 15.10 to 17.00 min for re-equilibration.

The flow rate was 0.30 mL/min and the injection volume was 3 μL. The column thermostatic oven was kept at 40 °C. The working conditions and parameters of the MS were optimized as follows: the ion source was ESI, operating in positive mode; the resolution of the precursor ion selector (Q-1) and product ion selector (Q-3) was 0.7 ± 0.1 amu; the curtain gas, ion source gas 1, and ion source gas 2 were set at 25, 45, and 10 psi, respectively; the source temperature was 550 °C; the ionization voltage was 5500 eV; and the entrance potential was 10 eV. MS acquisition was performed by Multiple Reaction Monitoring (MRM) mode. The MRM conditions and parameters including ion transitions, declustering potential (DP), and relative collision energy (CE) are provided in Table 1. The data acquisition and processing were performed using Analyst1.6.2 and MultiQuant2.1.1 software (Sciex, Darmstadt, Germany), respectively.

**Table 1:** MRM Parameters: Retention Time (RT), Precursor (Q-1) and Product (Q-3) Ion Transitions, Declustering Potential (DP), and Collision Energy (CE)

Compound	RT (min)	Q-1 (m/z)	Q-3 (m/z)	DP (eV)	CE (eV)
CBC-1	5.73	315.0	193.0	61	29
CBC-2		315.0	259.0	61	21
CBCA-1	2.49	359.2	219.1	50	27
CBCA-2		341.1	219.1	50	25
CBD-1	2.82	315.5	193.3	100	32
CBD-2		315.5	123.5	100	45
CBD-3		315.5	135.2	100	28
CBD2OAc-1	5.83	399.3	339.4	80	17
CBD2OAc-2		399.3	357.6	80	17
CBD2OAc-3		399.3	297.7	80	25
CBDA-1	2.48	359.2	219.2	81	41
CBDA-2		359.2	341.2	81	21
CBDV-1	1.94	287.0	165.0	80	23
CBDV-2		287.0	231.0	80	19
CBDVA-1	1.79	331.1	191.1	81	41
CBDVA-2		331.1	313.2	81	19
CBG-1	2.72	317.0	193.0	91	23
CBG-2		317.0	123.0	91	43
CBGA-1	2.65	361.2	219.1	76	37
CBGA-2		361.2	343.2	76	17
CBN-1	3.93	311.0	293.0	106	25
CBN-2		311.0	223.0	106	29
CBNA-1	5.05	355.5	235.1	50	25
CBNA-2		355.5	253.1	50	23
CBNOAc-1	6.81	353.3	311.5	80	18
CBNOAc-2		353.3	223.0	80	38
CBNOAc-3		353.3	293.0	80	30
Δ^9^-THCV-1	2.96	287.0	165.0	86	31
Δ^9^-THCV-2		287.0	123.0	86	43
Δ^9^-THCVA-1	3.78	331.1	191.1	71	43
Δ^9^-THCVA-2		331.1	313.2	71	21
Δ^8^-THC-1	4.73	315.5	193.0	100	32
Δ^8^-THC-2		315.5	259.0	100	20
Δ^8^-THC-3		315.5	123.5	100	45
Δ^8^-THCA-1	6.45	359.2	219.2	91	43
Δ^8^-THCA-2		359.2	341.2	91	21
Δ^8^-THCOAc-1	8.73	357.3	315.1	80	20
Δ^8^-THCOAc-2		357.3	193.1	80	40
Δ^8^-THCOAc-3		357.3	123.0	80	50
Δ^8^-THCP-1	8.22	343.3	221.3	80	30
Δ^8^-THCP-2		343.3	287.4	80	27
Δ^8^-THCP-3		343.3	123.1	80	47
Δ^8^-THCPOAc-1	13.17	385.2	343.4	80	21
Δ^8^-THCPOAc-2		385.2	221.1	80	40
Δ^8^-THCPOAc-3		385.2	123.0	80	55
Δ^9^-THC-1	4.58	315.5	193.0	100	32
Δ^9^-THC-2		315.5	259.0	100	20
Δ^9^-THC-3		315.5	123.5	100	45
Δ^9^-THCA-1	6.12	359.2	219.2	91	43
Δ^9^-THCA-2		359.2	341.2	91	21
Δ^9^-THCOAc-1	9.23	357.3	315.1	80	20
Δ^9^-THCOAc-2		357.3	193.1	80	40
Δ^9^-THCOAc-3		357.3	123.0	80	50
Δ^9^-THCP-1	7.96	343.3	221.3	80	30
Δ^9^-THCP-2		343.3	287.4	80	27
Δ^9^-THCP-3		343.3	123.1	80	47
9*R*-HHC-1	5.73	317.1	193.0	80	33
9*R*-HHC-2		317.1	137.0	80	30
9*R*-HHC-3		317.1	123.0	80	50
9*R*-HHCOAc-1	10.33	359.3	317.1	80	22
9*R*-HHCOAc-2		359.3	193.1	80	45
9*R*-HHCOAc-3		359.3	137.0	80	35
9*R*-HHCP-1	9.57	345.0	221.0	80	35
9*R*-HHCP-2		345.0	137.0	80	35
9*R*-HHCPOAc-1	13.90	387.0	345.0	80	35
9*R*-HHCPOAc-2		387.0	221.0	80	35
9*S*-HHC-1	5.56	317.1	193.0	80	33
9*S*-HHC-2		317.1	137.0	80	30
9*S*-HHC-3		317.1	123.0	80	50
9*S*-HHCOAc-1	9.85	359.3	317.1	80	22
9*S*-HHCOAc-2		359.3	193.1	80	45
9*S*-HHCOAc-3		359.3	137.0	80	35
9*S*-HHCP-1	9.27	345.0	221.0	80	35
9*S*-HHCP-2		345.0	137.0	80	35
9*S*-HHCPOAc-1	13.68	387.0	345.0	80	35
9*S*-HHCPOAc-2		387.0	221.0	80	35
*Internal standards (ISs)*					
CBD-*d*_3_-1	2.81	318.5	196.3	100	32
CBD-*d*_3_-2		318.5	135.2	100	28
Δ^9^-THC-*d*_3_-1	4.55	318.5	196.3	100	32
Δ^9^-THC-*d*_3_-2		318.5	135.2	100	28
Δ^9^-THCA-*d*_3_-1	6.09	362.2	222.2	91	43
Δ^9^-THCA-*d*_3_-2		362.2	344.2	91	21

### 2.6 Validation Procedure

The method was fully validated according to the international recommendations for the validation of a new analytical method in forensic toxicology.^23^ Selectivity was evaluated using blank matrices, when available, and in the absence of a suitable blank matrix, by a standard-addition procedure at low, medium, and high concentration levels. Six-point calibration curves for each compound, with eight replicates per level, were generated from the peak area ratios of the analytes to the IS versus nominal analyte concentration, including zero as a calibration level without forcing the intercept through the origin. Linearity was assessed by ordinary least-squares regression and verified by lack-of-fit analysis of variance (ANOVA) using replicated calibration levels. Sensitivity was expressed in terms of LOD (Limit of Detection) and LOQ (Limit of Quantification). The LOQ was determined as the lowest concentration with values for precision and accuracy within ±20% and a signal-to-noise (S/N) ratio of the peak areas ≥10, whereas the LOD was defined as the lowest concentration with a S/N of the peak areas ≥3. Precision and accuracy of the method were determined through the analysis of duplicate Quality Control (QC) samples at low, medium, and high concentration levels in eight different days for each compound according to their calibration ranges. Precision and accuracy were determined by calculating the coefficient of variation (CV%) and the Bias% of the QC samples and they were considered satisfactory if Bias was ± 15% of the nominal value (20% near LOQ); precision was within 15% RSD (20% near LOQ). The stability of the analytes at low and high concentration levels was assessed by comparing the mass spectrometric responses obtained after repeated injections of calibrators stored under different conditions: at room temperature for 24 h, at 4 °C for 48 h, and at −20 °C for 48 h, 15 days, and 30 days. The matrix effect was evaluated by fortifying each matrix with standard analyte solutions at the LOQ and at three additional concentration levels (low, medium, and high), and comparing the results with those obtained in spiked solvent after subtraction of the endogenous contribution of each analyte. The matrix effect was considered satisfactory if it was <20%, calculated as the percentage difference between the signal in the spiked matrix (blank-corrected) and the signal in the spiked solvent. Extraction recovery was indirectly assessed through extraction repeatability of Δ^9^-THC, CBD, and Δ^9^-THCA by performing six independent extractions (CV% < 20%).

### 2.7 Molecular Structures

The structures of cannabinoid receptors CB1 and CB2 were obtained from the Protein Data Bank (https://www.rcsb.org/) in complex with agonists (PDB: 5XRA^24^ and 5ZTY,^25^ respectively). The structures of Δ^9^-THC and 9*S*-HHC were obtained from the NIH PubChem repository (https://pubchem.ncbi.nlm.nih.gov/), while the structures of Δ^8^-THC, Δ^8^-THCOAc, Δ^8^-THCP, Δ^8^-THCPOAc, Δ^9^-THCOAc, Δ^9^-THCP, Δ^9^-THCPOAc, 9*S*-HHCOAc, 9*S*-HHCP, 9*S*-HHCPOAc, 9*R*-HHC, 9*R*-HHCOAc, 9*R*-HHCP, and 9*R*-HHCPOAc were manually edited starting from the downloaded structures using PyMOL.^26^

### 2.8 Molecular Protein–Ligand Docking

Docking simulations were conducted using AutoDock 4.2.6, a software suite designed to predict interactions between macromolecules and small ligands.^27^ The atomic charges, torsional flexibility, and protonation states of the enzyme and of the ligands were set up using, respectively, the AutoDock Tools graphical interface and Meeko (https://meeko.readthedocs.io). The ligand binding site for performing the docking calculation was identified on the basis of the ligand’s position in the original PDB structure, i.e., its geometric center was used to center the docking box for all the systems considered in the present work. The docking grid was set to 50 Å × 50 Å × 50 Å along the *x*, *y*, and *z* axes, with a grid spacing of 0.403 Å. Docking simulations employed a genetic algorithm, with each configuration file corresponding to a single docking run, generating up to 100 possible binding poses. These poses were then statistically analyzed based on their predicted dissociation constants (*K*_i_) expressed in terms of ligand concentration.

## 3 Results and Discussion

### 3.1 Cannabinoid Acetates Synthesis

At the time this study began, authentic reference standards of major cannabinoid acetates, including acetylated Δ^8^-THC, Δ^9^-THC, and HHC epimers, were not commercially available. Therefore, these compounds were synthesized in accordance with the procedures described in the Materials and Methods section (Section 2.2). Unlike earlier studies,^15,16^ where acetylation of cannabinoids was primarily used as a simple derivatization step without purification, the methodology adopted in this work included isolation of the acetylated products via flash column chromatography, followed by full structural characterization by NMR spectroscopy. This purification step was critical to remove residual reagents and byproducts, ensuring sufficient purity for comprehensive spectroscopic analysis.

Acetylation was carried out using acetic anhydride and triethylamine in dichloromethane, starting from milligram-scale quantities of pure precursors. TLC monitoring indicated complete conversion within 1 h at 50 °C, with no significant degradation or side reactions observed. Purification by silica gel flash chromatography using a solvent mixture of hexane/ethyl acetate yielded clean fractions of the acetylated products. Notably, the acetylation of the diastereomeric mixture of *9R*- and *9S*-HHC, affording the corresponding acetates, proceeded without epimerization at C-9, as confirmed by the retention of the original diastereomeric ratio.

The successful formation of the acetate esters was clearly supported by diagnostic NMR signals in CDCl_3_ (Supporting Information S1–S4). In the ^1^H NMR spectra, the appearance of a characteristic singlet corresponding to the methyl group of the acetate moiety (δ ≈ 2.28–2.32 ppm, 3H) confirmed acetylation of the phenolic hydroxyl group. Moreover, the disappearance of the phenolic OH signal observed in the starting materials further supported complete esterification. In the ^13^C NMR spectra, the presence of the ester carbonyl resonance (δ ≈ 169 ppm) together with the acetate methyl carbon signal (δ ≈ 20–21 ppm) provided additional confirmation of acetate formation.

Comprehensive structural characterization was achieved using both one- and two-dimensional NMR experiments (COSY, HSQC, and HMBC), allowing unambiguous assignment of all proton and carbon resonances. Overall, the synthetic and analytical strategy described herein provides purified and fully characterized cannabinoid acetate standards, addressing the limitations of reports that relied on unpurified derivatization products or incomplete spectroscopic data.

### 3.2 Method Validation

The LC-MS/MS method was developed and fully validated for the identification and quantitation of phytocannabinoids and SSCs as follows.

#### 3.2.1 Chromatographic Separation

In recent years, several techniques for the analysis of*Cannabis*-derived products have increasingly been tested. The two most common analytical methods are based on gas^11,28–30^ and liquid^31,32^ chromatography (GC and LC); however, high temperatures in the GC injection port turn carboxylate forms and, partially, acetylated forms (as shown in Supporting Information Figure S5) into their respective nonacidic or nonester forms, thus in the analytical result the specific contribution of each of them remains unknown. As a result, the detection and the quantitation of Δ^9^-THC do not necessarily imply that it was already present in the original sample but rather derived from the conversion of the carboxylate precursor Δ^9^-THCA and/or Δ^9^-THCOAc. To determine Δ^9^-THC and Δ^9^-THCA separately, a derivatization step prior to GC analysis is needed. Moreover, Cheng and Kerrigan suggested that GC liner and inlet conditions can influence the *in situ* formation of Δ^9^-THC from CBD cyclization.^33^ Contrarily, LC is the most effective and versatile technique for the analysis of complex mixtures of cannabinoids without derivatization, since it prevents decarboxylation of acidic forms, and it allows a separate identification and quantification of the neutral forms and their acidic or ester precursors. However, we found that a minor quantity of the acetylated form was de-esterified in the ion source of the LC-MS/MS system, when its temperature was set around 500 °C. We demonstrated that the acetylated forms that were converted in nonester forms were directly proportional to their initial concentration (Supporting Information Figure S6). Moreover, since the conversion occurs in the ion source, after the chromatographic separation, contrary to GC analysis, the contribution of the Δ^9^-THCOAc-derived Δ^9^-THC shows a different RT rather than the original Δ^9^-THC. Numerous studies, including those reviewed by La Maida et al.,^34^ have explored the chromatographic analysis of Δ^9^-THC, Δ^8^-THC, and other THC isomers across diverse matrices, noting their highly similar behavior. GC-MS effectively differentiates various THC isomers, while 9*R*- and 9*S*-HHC epimers separate readily via TLC, GC (native or TMS-derivatized), or LC^13,35,36^ . Holt et al.^16^ quantified Δ^8^-, Δ^9^-, and Δ^10^-THCOAc in cannabis products using GC-MS, and Tanaka et al.^14^ achieved partial Δ^8^- and Δ^9^-THCOAc separation by LC-PDA-MS. To our knowledge, this study introduces the first validated LC-MS/MS method that satisfactorily resolves, identifies, and quantifies multiple epimeric and isomeric SSC forms—including Δ^8^- and Δ^9^-THCOAc, Δ^8^- and Δ^9^-THCPOAc, 9*R*- and 9*S*-HHCOAc, and 9*R*- and 9*S*-HHCPOAc. Chromatographic separation is illustrated in Figure 6, achieved on a C18 column with an ACN-based mobile phase. A 17 min step gradient ensured baseline resolution of early-eluting cannabinoid acids, neutral cannabinoids, and late-eluting acetylated derivatives, with no methanol-related interferences observed.

**Figure 6:**
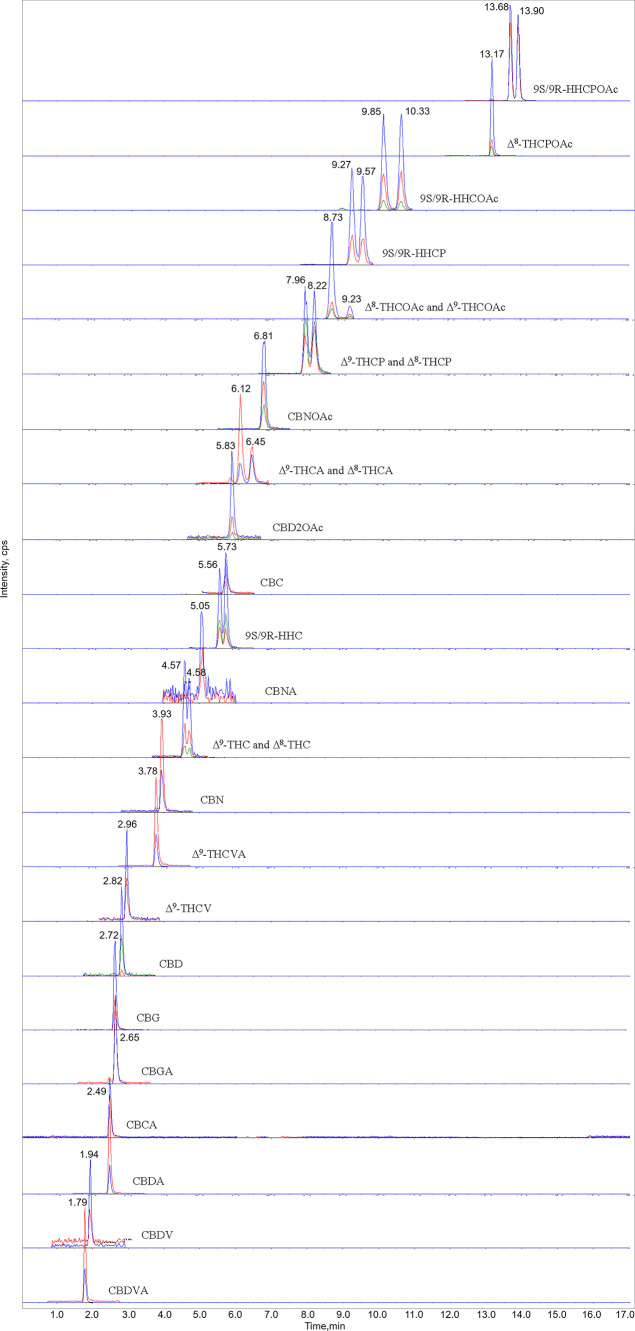
MRM chromatogram of phytocannabinoids and SSCs standard mixture at an intermediate concentration level.

#### 3.2.2 Selectivity, Linearity, LOD, and LOQ

Across all analytes and matrices, no additional coeluting peaks were observed. When a blank matrix was unavailable, the chromatographic peaks of endogenous and spiked analytes showed comparable retention times and ion ratios. For each analyte, the ordinary linear model showed no statistically significant lack of fit over the investigated range (*p* > 0.05). The mean %CV, LOD, and LOQ are listed in Table 2.

**Table 2:** Calibration Parameters for Selected Analytes

Analyte	Calibration range (%)	Calibration range (ng/mL)	CV %	LOD (ng/mL)	LOQ (ng/mL)
Δ^8^-THC	1–50^a^	0.5–25^a^	14	0.08	0.25
Δ^8^-THCA			12	0.01	0.03
Δ^9^-THC			15	0.05	0.17
Δ^9^-THCA			13	0.01	0.04
CBC	0.1–2.5^b^	1 – 25^b^	8.4	0.06	0.21
CBCA			11	0.02	0.07
CBD			8.4	0.04	0.12
CBDA			7.1	0.01	0.05
CBDV			9.1	0.05	0.16
CBDVA			5.0	0.01	0.04
CBG			6.9	0.02	0.05
CBGA			7.8	0.01	0.05
CBN			8.4	0.06	0.19
CBNA			8.0	0.13	0.42
Δ^9^-THCV			11	0.18	0.61
Δ^9^-THCVA			5.1	0.01	0.04
9*R*-HHC			8.0	0.03	0.11
9*S*-HHC			7.3	0.03	0.11
Δ^9^-THCOAc	0.001–0.025^c^	1 – 25^c^	6.3	0.04	0.14
CBD_2_OAc	0.001–0.05^c^	1 – 50^c^	9.9	0.05	0.17
CBNOAc			8.4	0.02	0.08
Δ^8^-THCOAc			7.3	0.01	0.03
Δ^8^-THCP			7.5	0.04	0.13
Δ^8^-THCPOAc			7.0	0.01	0.04
Δ^9^-THCP			7.7	0.02	0.07
9*R*-HHCOAc			6.1	0.02	0.05
9*R*-HHCP			7.5	0.04	0.13
9*R*-HHCPOAc			7.2	0.01	0.03
9*S*-HHCOAc			7.4	0.02	0.05
9*S*-HHCP			6.9	0.03	0.08
9*S*-HHCPOAc			8.5	0.01	0.03

a 50 ng/mL was set as 100%.

b 1 μg/mL was set as 100%.

c 100 μg/mL was set as 100%.

#### 3.2.3 Inter-Day Precision and Accuracy

The method showed good performance, with CV values ≤ 15% (≤20% at the LOQ) for precision and bias ≤ 15.6% for accuracy. Precision and accuracy data for all analytes are reported in Table 3.

**Table 3:** Precision and Accuracy for Each Analyte at Low, Medium, and High Concentration Level

Analyte	Concentration level (%)	Precision (%)	Accuracy (%)
Δ^8^-THC	1	12	3.2
	5	5.3	5.3
	50	0.82	–0.62
Δ^8^-THCA	1	18	–16
	5	7.8	12
	50	0.96	–0.84
Δ^9^-THC	1	7.1	16
	5	9.9	6.5
	50	1.3	0.02
Δ^9^-THCA	1	10	–6.8
	5	5.6	5.7
	50	0.25	–0.15
CBC	0.1	17	–3.9
	0.25	11	4.3
	2.5	2.3	–0.79
CBCA	0.1	7.1	–1.1
	0.5	3.1	0.98
	2.5	0.16	0.02
CBD	0.1	4.9	0.92
	0.5	5.8	2.9
	2.5	1.3	–0.60
CBDA	0.1	8.4	–7.2
	0.5	3.7	0.75
	2.5	0.48	0.19
CBDV	0.1	17	–17
	0.5	9.2	5.04
	2.5	1.2	–0.80
CBDVA	0.1	11	–9.0
	0.5	4.6	1.4
	2.5	0.43	0.32
CBG	0.1	18	–7.3
	0.5	7.0	2.2
	2.5	2.4	–0.75
CBGA	0.1	14	–10
	0.5	4.3	1.9
	2.5	0.42	0.09
CBN	0.1	7.6	–6.3
	0.5	6.7	3.0
	2.5	1.3	–0.79
CBNA	0.1	13	1.9
	0.5	3.4	1.2
	2.5	0.67	0.02
Δ^9^-THCV	0.1	6.9	–9.0
	0.5	13.2	2.8
	2.5	0.21	0.05
Δ^9^-THCVA	0.1	8.2	–7.8
	0.5	1.4	0.87
	2.5	0.52	0.33
9*R*-HHC	0.1	18	–1.3
	0.5	6.3	6.8
	2.5	1.63	–0.55
9*S*-HHC	0.1	8.8	–11
	0.5	9.8	4.0
	2.5	1.3	–0.28
Δ^9^-THCOAc	0.001	18.7	–1.7
	0.005	6.0	1.7
	0.025	1.3	0.20
CBD2OAc	0.001	20	–6.7
	0.005	7.4	3.0
	0.05	2.0	0.63
CBNOAc	0.001	12	–6.0
	0.005	5.2	–0.4
	0.05	1.9	0.08
Δ^8^-THCOAc	0.001	15	–5.0
	0.005	5.6	3.0
	0.05	1.7	0.77
Δ^8^-THCP	0.001	17	–12
	0.005	7.7	0.7
	0.05	1.3	0.03
Δ^8^-THCPOAc	0.001	19	–10
	0.005	7.7	4.0
	0.05	1.9	0.60
Δ^9^-THCP	0.001	11	–8.3
	0.005	5.4	2.3
	0.05	1.3	0.60
9*R*-HHCOAc	0.001	12	–10
	0.005	5.3	4.0
	0.05	1.5	0.7
9*R*-HHCP	0.001	6.0	–13
	0.005	4.2	4.0
	0.05	1.4	–0.10
9*R*-HHCPOAc	0.001	18	–12
	0.005	5.2	0.00
	0.05	2.0	0.30
9*S*-HHCOAc	0.001	19	–10
	0.005	5.7	3.3
	0.05	1.6	0.33
9*S*-HHCP	0.001	18	–12
	0.005	8.7	1.7
	0.05	1.7	0.07
9*S*-HHCPOAc	0.001	19	–10
	0.005	5.8	3.7
	0.05	2.3	0.97

#### 3.2.4 Stability

Calibrators stability was assessed by monitoring the percentage deviation of each analyte in the vial over time. After 24 h of storage at room temperature, deviations ranged from 1.00% to 15.68%, except for 9*R-* and 9*S*-HHCPOAc (<30%). After 48 h, deviations ranged from 2.45% to 15.92% at 4 °C (except for 9*R* and 9*S*-HHCPOAc (<40%)) and from 3.13% to 15.54% at −20 °C. Long-term calibrator stability was assessed exclusively at −20 °C obtaining a deviation ranging from 2.43% to 15.57% after 15 days and from 5.16% to 15.78% after 30 days.

#### 3.2.5 Matrix Effect and Extraction Repeatability

Across all analytes and matrices, the matrix effect was within the predefined acceptance limits, ranging from 0–22% at the LOQ and low level, 0–19% at the medium level, and 0–16% at the high level, thus confirming the suitability of solvent-based calibration for quantitative purposes. Regarding extraction repeatability, the coefficients of variation for Δ^9^-THC, CBD, and Δ^9^-THCA obtained from six replicate extractions were consistently below 20% for all matrices, indicating acceptable and reproducible extraction performance. Considering the close structural similarity among the investigated analytes, these percentage values can reasonably be assumed to be representative for all compounds included in the method.

### 3.3 Analysis of Seized*Cannabis* *-*Derived Samples

The validated LC-MS/MS method was subsequently applied to characterize phytocannabinoid SSCs in 151 cannabis-derived products seized between 2024 and 2025 in Northern Italy. The samples primarily consisted of cannabis flowers (45.7%, *n* = 69) and resins (45.7%, *n* = 69), while vape liquids (6.0%, *n* = 9), waxes (2.0%, *n* = 3), and powder (0.7%, *n* = 1) represented a smaller proportion.

An overview of the frequency of detection of all cannabinoids is reported in Table 4, which summarizes the complete quantitative data set. Δ^9^-THC and Δ^9^-THCA were detected in almost all samples, while Δ^9^-THCOAc (85%), Δ^9^-THCP (61%), Δ^8^-THC (18%), and Δ^8^-THCOAc (6%) were the most frequently identified SSCs.

**Table 4:** Quantitative Results of Main Phytocannabinoids and SSCs

Analyte	Product type	Number	Mean (%)	Median (%)	Range (%)
Δ^9^-THC	Flowers	65	3.73	3.07	0.13–14.11
	Resin	66	10.74	10.17	0.17–26.04
	Waxes	1	70.10	70.10	70.10
	Vape liquids	9	12.97	6.44	1.19–38.77
Δ^9^-THCA	Flowers	69	12.89	11.37	0.05–37.86
	Resin	68	14.27	10.79	0.09–48.14
	Waxes	1	0.13	0.13	0.13
	Vape liquids	4	0.44	0.40	0.28–0.70
	Powder	1	88.00	88.00	88.00
CBD	Flowers	40	1.00	0.05	0.01–10.75
	Resin	69	3.71	0.32	0.03–63.69
	Waxes	2	80.67	80.67	63.84–97.50
	Vape liquids	7	0.31	0.38	0.08–0.66
CBDA	Flowers	55	0.92	0.05	0.005–15.84
	Resin	69	1.01	1.03	0.05–5.90
CBN	Flowers	54	0.29	0.15	0.03–3.31
	Resin	68	0.64	0.46	0.04–4.47
	Waxes	1	0.82	0.82	0.82
	Vape liquids	9	1.99	0.62	0.32–12.49
Δ^8^-THC	Resin	18	2.85	2.65	1.91–7.18
	Vape liquids	9	33.22	43.72	0.92–53.72
9*R*-HHC	Flowers	2	1.18	1.18	0.89–1.47
	Vape liquids	5	5.26	1.25	0.22–20.99
9*S*-HHC	Flowers	2	0.87	0.87	0.67–1.07
	Vape liquids	4	2.91	0.53	0.18–10.40
Δ^9^-THCP	Flowers	31	0.004	0.0009	0.0004–0.03
	Resin	56	0.002	0.002	0.0001–0.004
	Vape liquids	5	0.0007	0.0008	0.0001–0.001
Δ^8^-THCP	Flowers	3	0.08	0.08	0.07–0.09
	Vape liquids	3	0.002	0.002	0.001–0.002
9*R*-HHCP	Flowers	2	0.40	0.40	0.29–0.51
	Vape liquids	1	0.004	0.004	0.004
9*S*-HHCP	Flowers	2	0.04	0.04	0.03–0.05
	Vape liquids	1	0.0008	0.0008	0.0008
Δ^9^-THCOAc	Flowers	58	0.0008	0.0004	0.0001–0.008
	Resin	67	0.11	0.0008	0.0001–6.99
	Waxes	1	0.0009	0.0009	0.0009
	Vape liquids	2	0.0004	0.0004	0.0003–0.0005
Δ^8^-THCOAc	Flowers	2	0.02	0.02	0.0002–0.03
	Resin	1	0.63	0.63	0.63
	Vape liquids	6	0.001	0.001	0.0004–0.002
9*R*-HHCOAc	Flowers	3	3.35	3.44	0.002–6.62
9*S*-HHCOAc	Flowers	3	1.94	2.11	0.001–3.72
9*R*-HHCPOAc	Flowers	2	0.003	0.003	0.002–0.003
9*S*-HHCPOAc	Flowers	2	0.0008	0.0008	0.0004–0.001

#### 3.3.1 Cannabis Flowers

Δ^9^-THCA was detected in 100% of flower samples, while Δ^9^-THC was also present in nearly all samples; concentrations ranged from 0.05% to 37.86% (mean: 12.89%; median: 11.37%) for Δ^9^-THCA and 0.13–14.11% (mean: 3.73%; median: 3.07%) for Δ^9^-THC and overall Δ^9^-THCA exceeded Δ^9^-THC in 90% of samples, confirming that acidic cannabinoids predominate in plant material.

Δ^9^-THCOAc was detected in 84% of samples at trace levels (range: LOQ–0.008%; median: 0.0004%). Δ^9^-THCP was also frequently observed at low concentrations (range: LOQ–0.03%; median: 0.0009%).

Δ^8^-THC was not detected in flower samples; however, Δ^8^-THCOAc was identified in two samples (0.02%), which also contained multiple hydrogenated cannabinoids (HHCs and derivatives), suggesting possible exogenous addition.

#### 3.3.2 Resins

Δ^9^-THCA and Δ^9^-THC were detected in 99% of resin samples with Δ^9^-THCA ranging from 0.09% to 48.14% (mean: 14.27%; median: 10.79%), and Δ^9^-THC from 0.17% to 26.04% (mean: 10.74%; median: 10.17%); Δ^9^-THCA exceeded Δ^9^-THC in 58% of samples, whereas the opposite trend was observed in 42%, indicating partial decarboxylation or processing and Δ^8^-THC was frequently associated with samples where Δ^9^-THC > Δ^9^-THCA, being quantified above the LOQ in 18 samples (mean ∼3%), suggesting potential manipulation or addition of a synthetic product, although trace Δ^8^-THC was also detected in additional samples.

Δ^9^-THCOAc was highly prevalent (range: LOQ–6.99%; median: 0.0008%), supporting the hypothesis of either natural occurrence at trace levels or intentional addition at higher concentrations, and Δ^9^-THCP was also commonly detected at low concentrations (range: LOQ–0.0041%; median: 0.0019%).

Some resin samples exhibited unusually high CBD contents (up to 63.7%), consistent with CBD-rich chemotypes. One such sample also contained significant levels of Δ^9^-THCOAc (7.0%), Δ^8^-THC (2.1%), and Δ^8^-THCOAc (0.6%), while Δ^9^-THC and Δ^9^-THCA were, respectively, 1.3 and 0.1% indicating a complex and possibly altered composition.

#### 3.3.3 Vape Liquids

Δ^9^-THCA was detected in 44% of vape liquids, while Δ^9^-THC was more consistently present; Δ^8^-THC was identified in all nine samples, with concentrations reaching up to 54%.

Δ^8^-THCOAc was detected in six samples (median: 0.001%), and in three cases co-occurred with Δ^8^-THCP > LOQ, suggesting synthetic formulation.

Δ^9^-THCP was also detected at trace levels (range: 0.0001–0.0012%; median: 0.0008%) in 55% of the samples.

#### 3.3.4 Waxes and Powder

None of the wax samples contained SSCs. However, one sample exhibited the highest Δ^9^-THC concentration observed (70.1%), while the remaining two showed extremely high CBD contents (97.5 and 63.8%).

The single powder sample contained Δ^9^-THCA (88% detection), although no further SSCs were identified.

### 3.4 Structure–Activity Relationship of SSCs

We have also investigated the structure–activity relationship (SAR) of SSCs by means of molecular docking models, which have been computed with the aim of evaluating the entity of their binding to CB1 and CB2. CB1 is the main target of the present study, and the latter serves as a control due to their similar fold but with a different THC orientation in the binding pocket. Binding affinity was evaluated through multiple (100) runs of the docking calculations for a series of ligands among the ones presented above and computing the distribution of the dissociation constants (namely *K*_i_) over all the docking poses. This metric reflects the strength of interaction between an inhibitor and its target: a shift of the distributions toward lower *K*_i_ values clearly indicates stronger interaction (i.e., a lower *K*_i_ corresponds to a lower free Gibbs energy of binding). The distributions of the computed *K*_i_ of the derivatives of Δ^8^-THC, Δ^9^-THC, 9*R*-HHC, and 9*S*-HHC (Figure 7A) revealed in all cases an increase of the affinity toward CB1 as a response to the chemical modifications considered in the present work, i.e., an insertion of the acetate group or the hydrophobic side-chain extension. All the investigated compounds showed a dramatic increase in the binding affinity once the acetate group was introduced in the molecule, ranging from halved average *K*_i_ values for both THC forms (Δ^8^-THC and Δ^9^-THC) to a 10-fold decrease for HHC, whereas the elongated side-chain had a strong effect mainly on the binding of both THC derivatives, as previously suggested by Citti et al.^19^ (Δ^9^-THCP_*K*_i__ = 1.2 versus Δ^9^-THC_*K*_i__ = 40). Moreover, this study shows that 9*R* epimers of HHC and HHCP had stronger binding affinity than their 9*S* counterparts based on *K*_i_ values (Figure 7A), confirming that small structural modifications can alter pharmacological profiles.^7,12,17,18,37^ The role of the acetate group in increasing the stabilization of receptor–ligand interactions can be explained in our models by the formation of hydrogen bonds with neighboring residues His 178 and Ser 383 (Figure 7B: relevant information is reported only for Δ^9^-THC, for the other systems they can be found in Supporting Information Figures S7 and S8). Ser 383 stably interacts with all the molecules considered in the present study via its Oγ side chain atom (Figure S7), also supporting recently reported results^38,39^ about the role of these molecules as prodrugs upon acetate hydrolysis, i.e., they can efficiently bind to the receptor even in the absence of the acetate moiety and only due to the ubiquitous presence of O1. A role of further stabilization, with respect to molecules without the acetate functional group, can be envisaged for His 178 (Figure S8), as it stabilizes the complex via Nε2–O hydrogen bonding (roughly in 25% of the poses). Aside from hydrogen bonding, an elongated hydrophobic side-chain can more easily be accommodated by the hydrophobic binding pocket of the receptor, formed by residues Phe 268, Ile 271, Tyr 275, Leu 276, and Trp 279 (Figure 7C). This is in agreement with recently documented experimental findings about an increased potency (EC50) of compounds as a function of the length of the hydrophobic side-chain, i.e., Δ^9^- and Δ^8^-THC homologues with longer alkyl chains possess increased CB1 activation potential.^40^ The same trend in the energetic/inhibitory profiles has been observed for the receptor CB2 (Supporting Information Figure S9), with a more pronounced synergy between the two modifications: the distributions of *K*_i_ are broader, i.e., with a significant part of the *K*_i_ values higher than 200 nM, suggesting a less specific interaction with respect to the one with CB1.

**Figure 7:**
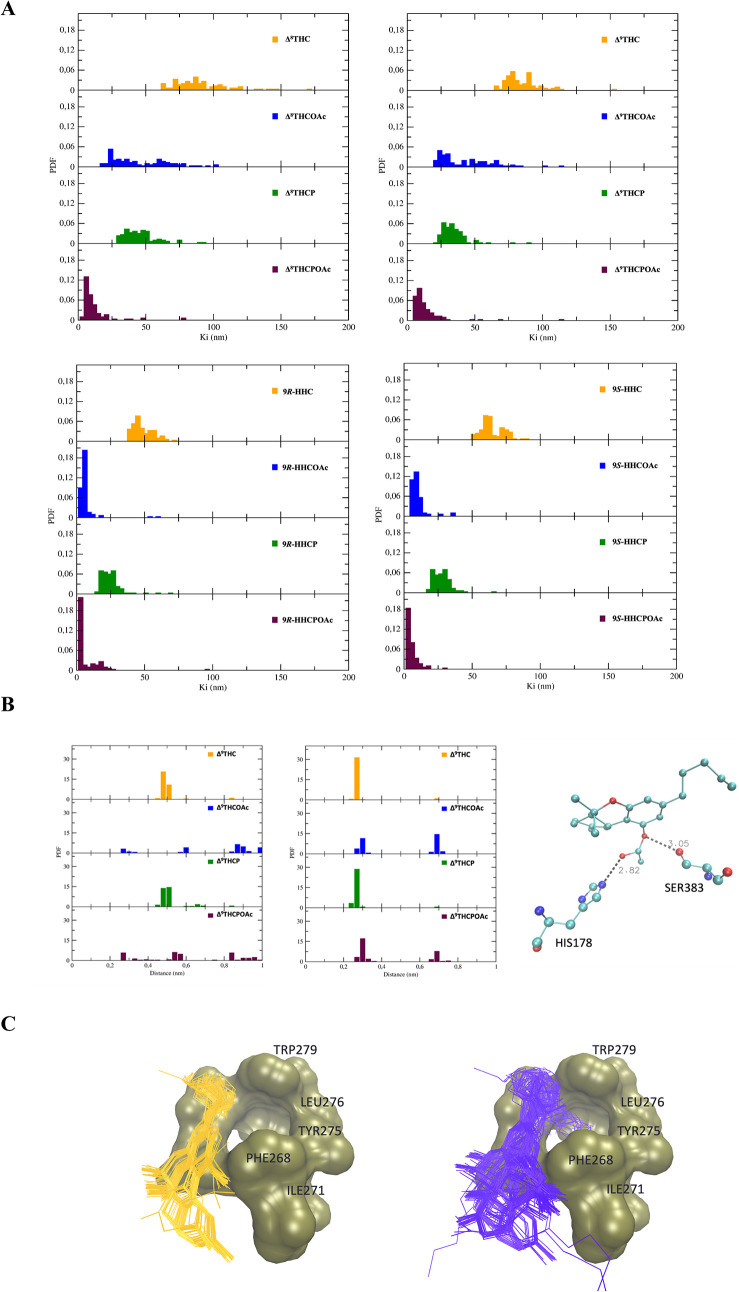
A) Probability distribution function (PDF) of the dissociation constants (*K*_i_), computed by AutoDock between THC derivatives and the CB1 receptor. From the top of each panel, the ligands are presented in the following order: native compound (Δ^8^-THC, Δ^9^-THC, 9*R*-HHC, and 9*S*-HHC), acetate group addition, side-chain extension, and simultaneous acetate group addition. B) Probability distribution function (PDF) of the distance between the His 178 Nε2 atom and the phenolic hydroxyl group O1 atom of Δ^9^-THC/Δ^9^-THCP or the carboxylic O atom of the acetate moiety of Δ^9^-THCOAc/Δ^9^-THCPOAc (left panel) and between the Ser 383 Oγ atom and the O1 atom of the phenolic hydroxyl group of Δ^9^-THC (right panel). A representative structure of these interactions is reported. C) Representation of the hydrophobic pocket of CB1, constituted by residues 193, 197, 279, 275, 276, 271, 268, and 363 (only partially displayed), and of the bundle of docking poses of the Δ^9^-THC (left, orange) and Δ^9^-THCPOAc (right, blue).

## 4 Conclusions

The synthetic strategy described herein provides purified and fully characterized cannabinoid acetate standards (Δ^8^-THCOAc, Δ^9^-THCOAc, 9*R-* and 9*S*-HHCOAc, and CBNOAc), thereby overcoming limitations of earlier reports that relied on unpurified derivatization products or incomplete spectroscopic characterization. The availability of purified and fully characterized cannabinoid acetate standards is particularly important in light of increasing analytical, forensic, and regulatory interest. The developed LC separation successfully resolved multiple epimeric and isomeric cannabinoid structures, and the validated LC-MS/MS method proved effective for the identification and quantification of both phytocannabinoids (including acidic and neutral forms) and SSCs in *Cannabis*-derived products. This analytical approach enabled the reliable identification and structural characterization of these compounds, contributing to the expanding knowledge base required for their detection and monitoring. The method presented here could therefore be implemented in routine analytical workflows to enable the detection of SSCs beyond those currently under regulatory control. The present study highlights the high prevalence of SSCs in *Cannabis*-derived products, posing a potential risk to consumers who are often unaware of the actual composition of the products they consume. Indeed, molecular docking analyses suggested a potentially increased binding propensity toward CB1 for acetylated and side-chain-extended derivatives, although these *in silico* findings require experimental confirmation. Overall, these findings underline the importance of continued analytical surveillance and further pharmacological investigation of newly emerging cannabinoid derivatives.

## Supplementary Material



## Notes

NMR spectra (Figures S3–S6); GC-MS chromatograms (Figures S7–S8); linear function between acetylated and acetylated-derived nonester forms by LC-MS/MS (Figures S9–S10); computational details (Figures S9–S12) (PDF)

## Abbreviations

CBC: Cannabichromene
CBCA: Cannabichromenic acid
CBD: Cannabidiol
CBD2OAc: Cannabidiol diacetate
CBDA: Cannabidiolic acid
CBDV: Cannabidivarin
CBDVA: Cannabidivarinic acid
CBG: Cannabigerol
CBGA: Cannabigerolic acid
CBN: Cannabinol
CBNA: Cannabinolic acid
CBNOAc: Cannabinol acetate
Δ^8^-THC: Delta-8-Tetrahydrocannabinol
Δ^8^-THCA: Delta-8-Tetrahydrocannabinolic acid
Δ^8–^THCOAc: Delta-8-Tetrahydrocannabinol acetate
Δ^8^-THCP: Delta-8-Tetrahydrocannabiphorol
Δ^8^-THCPOAc: Delta-8-Tetrahydrocannabiphorol acetate
Δ^9^-THC: Delta-9-Tetrahydrocannabinol
Δ^9^-THCA: Delta-9-Tetrahydrocannabinolic acid
Δ^9^-THCOAc: Delta-9-Tetrahydrocannabinol acetate
Δ^9^-THCP: Delta-9-Tetrahydrocannabiphorol
Δ^9^-THCV: Delta-9-Tetrahydrocannabivarin
Δ^9^-THCVA: Delta-9-Tetrahydrocannabivarinic acid
9*R*-HHC: (6aR,9R,10aR)-Hexahydrocannabinol
9*R*-HHCOAc: (6aR,9R,10aR)-Hexahydrocannabinol acetate
9*R*-HHCP: (6aR,9R,10aR)-Hexahydrocannabiphorol
9*R*-HHCPOAc: (6aR,9R,10aR)-Hexahydrocannabiphorol acetate
9*S*-HHC: (6aR,9S,10aR)-Hexahydrocannabinol
9*S*-HHCOAc: (6aR,9R,10aR)-Hexahydrocannabinol acetate
9*S*-HHCP: (6aR,9S,10aR)-Hexahydrocannabiphorol
9*S*-HHCPOAc: (6aR,9S,10aR)-Hexahydrocannabiphorol acetate
