# Supplementary material for: Comprehensive Profiling of Phytocannabinoids and Semisynthetic Cannabinoids in Seized Materials from Northern Italy
Source: ACS Omega. 2026 May 27;11(22):32548–61. doi: 10.1021/acsomega.6c00941 (PMC13261594; doi:10.1021/acsomega.6c00941)
Supplement: Supplementary file 1 [file ao6c00941_si_001.pdf]

Supporting Information

Comprehensive Profiling of Phytocannabinoids and Semi-Synthetic Cannabinoids in Seized Materials from Northern Italy

Sara Casati[1,2,*] Roberta F. Bergamaschi[1], Lea Sicuro[2,3], Alessandro Ravelli[1], Sofia Vanerio[1], Valentina Orsi[1], Camilla Ronco[1], Enrica Torretta[1], Alberto Rimoldi[1], Gabriella Roda[4], Alessio Battistini[1], Luca Mollica[3], Paola Rota[5,6]

[1] Laboratorio di Tossicologia Forense, Dipartimento di Scienze Biomediche, Chirurgiche e Odontoiatriche, Università degli Studi di Milano, Via Luigi Mangiagalli 37, 20133, Milan, Italy

[2] Fondazione IRCCS Ca' Granda Ospedale Maggiore Policlinico, Via Francesco Sforza 35, 20135, Milan, Italy, *sara.casati@unimi.it

[3] Dipartimento di Biotecnologie Mediche e Medicina Traslazionale, Università degli Studi di Milano, Via Fratelli Cervi, 93, 20054, Segrate, Italy

[4] Dipartimento di Scienze Farmaceutiche, Università degli Studi di Milano, Via Trentacoste 2, 20134, Milan, Italy

[5] Dipartimento di Scienze Biomediche, Chirurgiche e Odontoiatriche, Università degli Studi di Milano, Via Della Commenda, 20121, Milan, Italy

[6] Institute for Molecular and Translational Cardiology (IMTC), San Donato Milanese, 20097, Milan, Italy

Index	Page
**S1.** ^{1}H NMR spectrum of 9*R*- and 9*S*-HHCOAc epimeric mixture	S3
**S2.** ^{1}H and ^{13}C NMR spectra of Δ^{9}-THCOAc	S4
**S3.** ^{1}H and ^{13}C NMR spectra of Δ^{8}-THCOAc	S5
**S4.** ^{1}H and ^{13}C NMR spactra of CBNOAc	S6
**S5.** GC-MS chromatogram of Δ^{9}-THCOAc (A, RT 13.93), Δ^{8}-THCOAc (B, RT 13.31), 9*R*-HHCOAc (C, RT 12.83), 9*S*-HHCOAc (D, RT 13.31), Δ^{8}-THCPOAc (E, RT 15.09), 9*R*-HHCPOAc (F, RT 14.70) and 9*S*-HHCPOAc (G, RT 15.17) (1 µg/mL) showed the partial formation of Δ^{9}-THC (A, RT 13.46), Δ^{8}-THC (B, RT 13.70), 9*R*-HHC (C, RT 13.26), 9*S*-HHC (D, RT 13.35), and Δ^{8}-THCP (E, RT 15.58), 9*R*-HHCP (F, RT 15.17) and 9*S*-HHCP (G, RT 15.28)	S7-8
**S6.** Linear function (R>0.999) between Δ^{9}-THCOAc (A), Δ^{8}-THCOAc (B), 9*R*-HHCOAc (C), 9*S*-HHCOAc (D), Δ^{8}-THCPOAc (E), 9*R*-HHCPOAc (F) and 9*S*-HHCPOAc (G) concentrations and acetylated-derived non-ester forms by LC-MS/MS analysis.	S9-10
**S7.** Probability distribution function (PDF) of the distance between Ser 383 Oγ atom and the O1 atom of the phenolic hydroxyl group of THC derivatives.	S11
**S8.** Probability distribution function (PDF) of the distance between His 178 Nε2 atom and the O1 atom of the phenolic hydroxyl group of THC derivatives.	S12
**S9.** Probability distribution function (PDF) of the binding constant (Ki) computed by AutoDock between THC derivatives and the cannabinoid receptor 2 (CB2).*S13*	

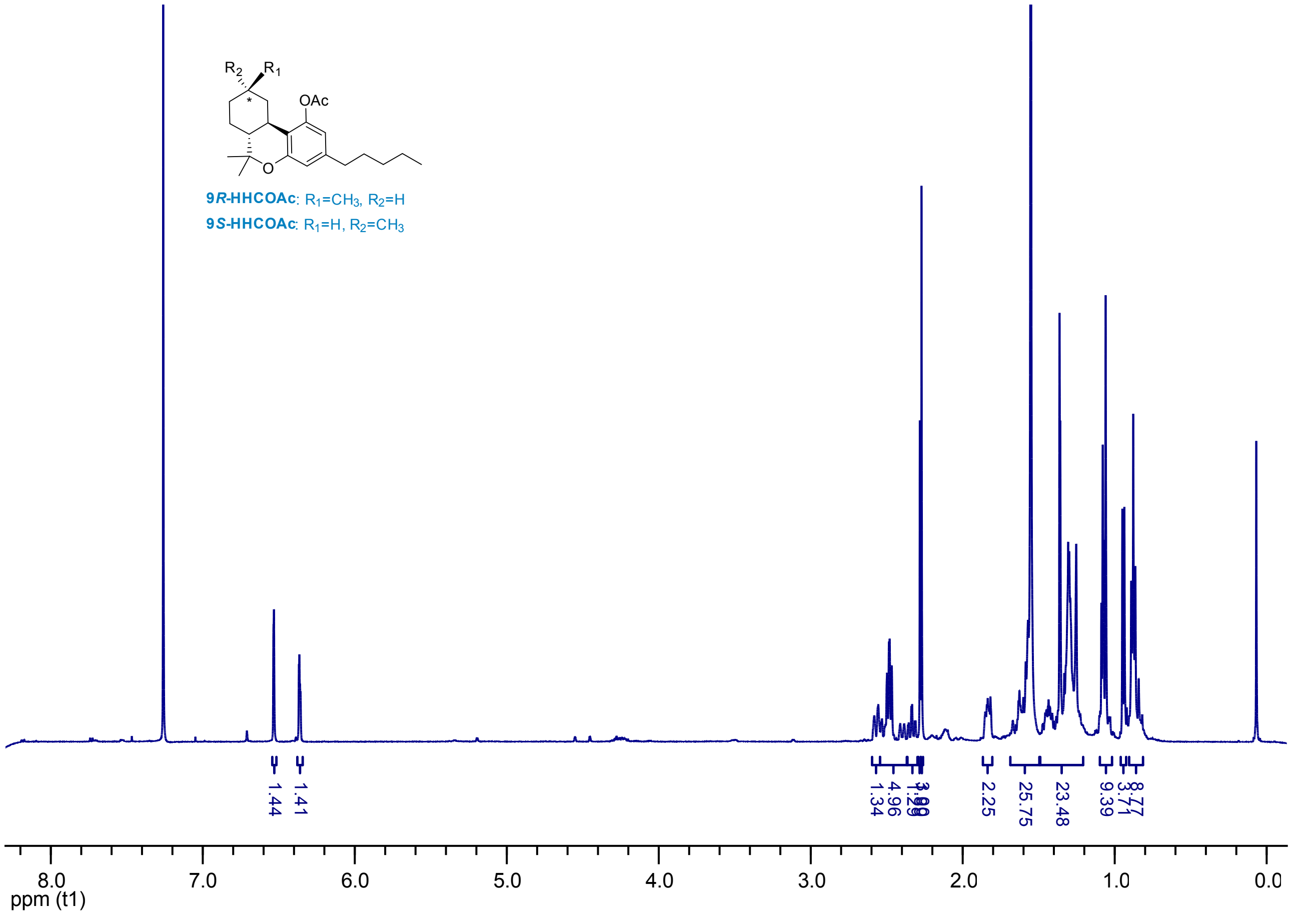
OAc
9*R*-HHCOAc: R_1=CH_3, R_2=H
9*S*-HHCOAc: R_1=H, R_2=CH_3
1.44
1.41
1.34
4.96
2.25
25.75
23.48
9.39
3.71
8.77
8.0
7.0
6.0
5.0
4.0
3.0
2.0
1.0
0.0
ppm (t1)

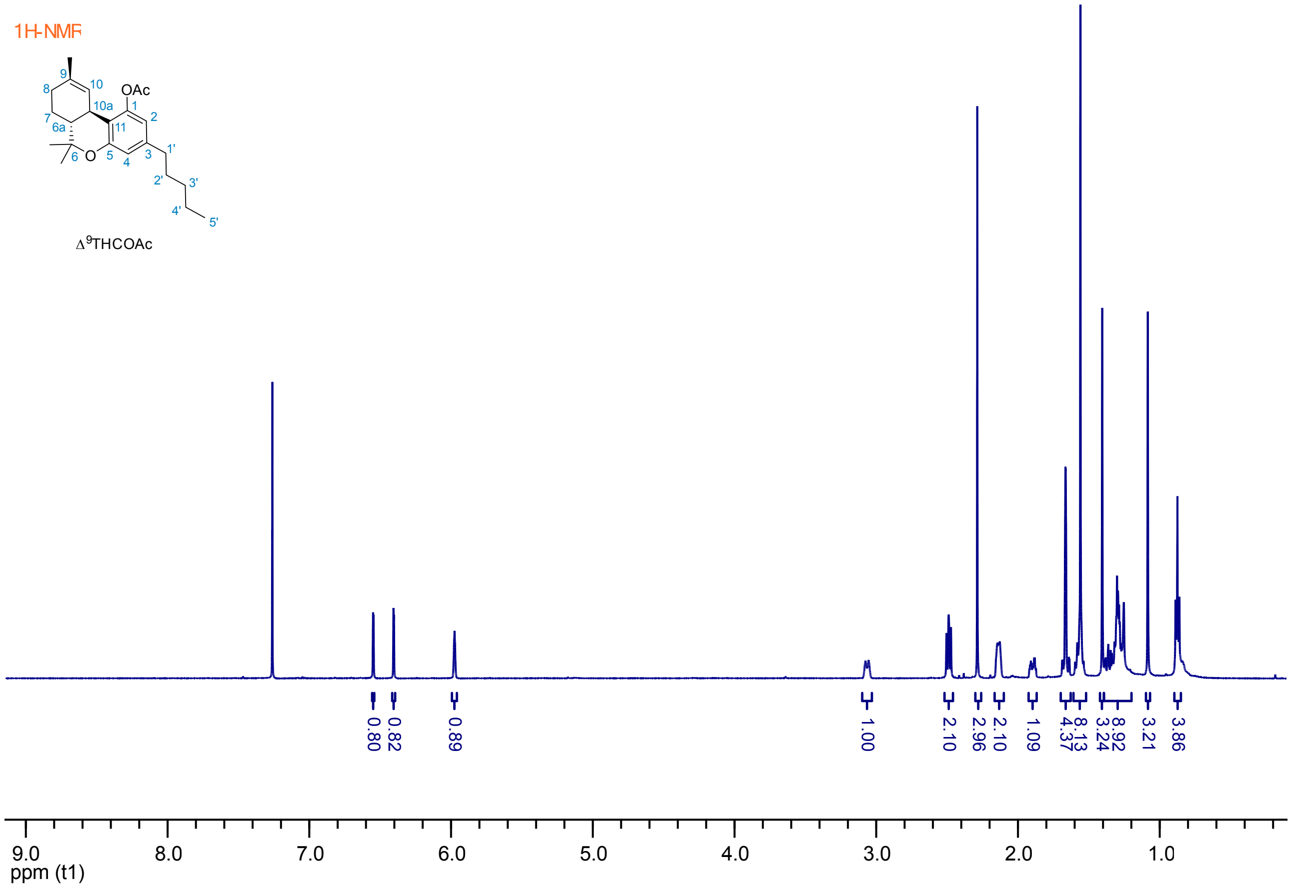
OAc
Δ9THCOAc
0.80 0.82 0.89 1.00 2.10 2.96 2.10 1.09 4.37 8.13 3.24 8.92 3.21 3.86
9.0 8.0 7.0 6.0 5.0 4.0 3.0 2.0 1.0
ppm (t1)

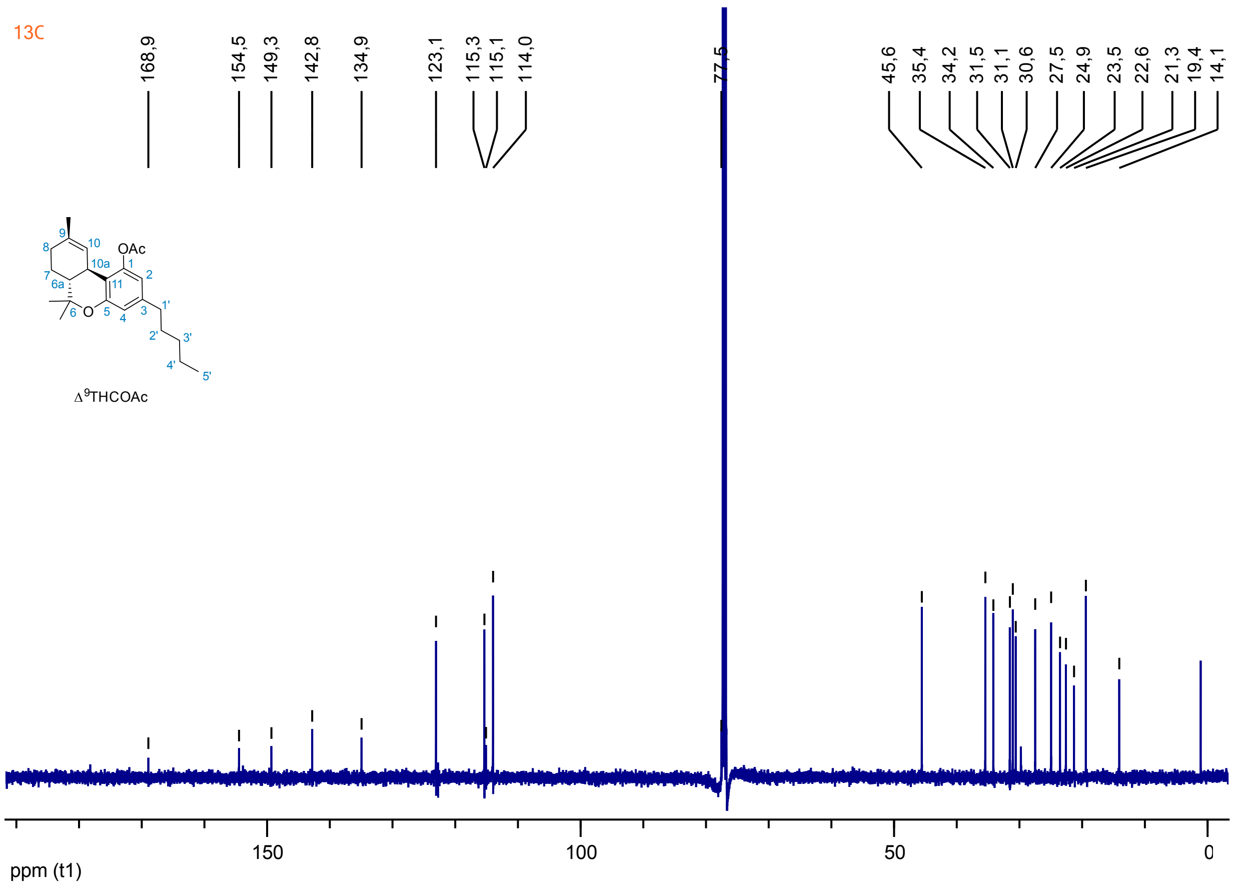
168,9 154,5 149,3 142,8 134,9 123,1 115,3 115,1 114,0
45,6 35,4 34,2 31,5 31,1 30,6 27,5 24,9 23,5 22,6 21,3 19,4 14,1
OAc
Δ9THCOAc
150 100 50 0
ppm (t1)

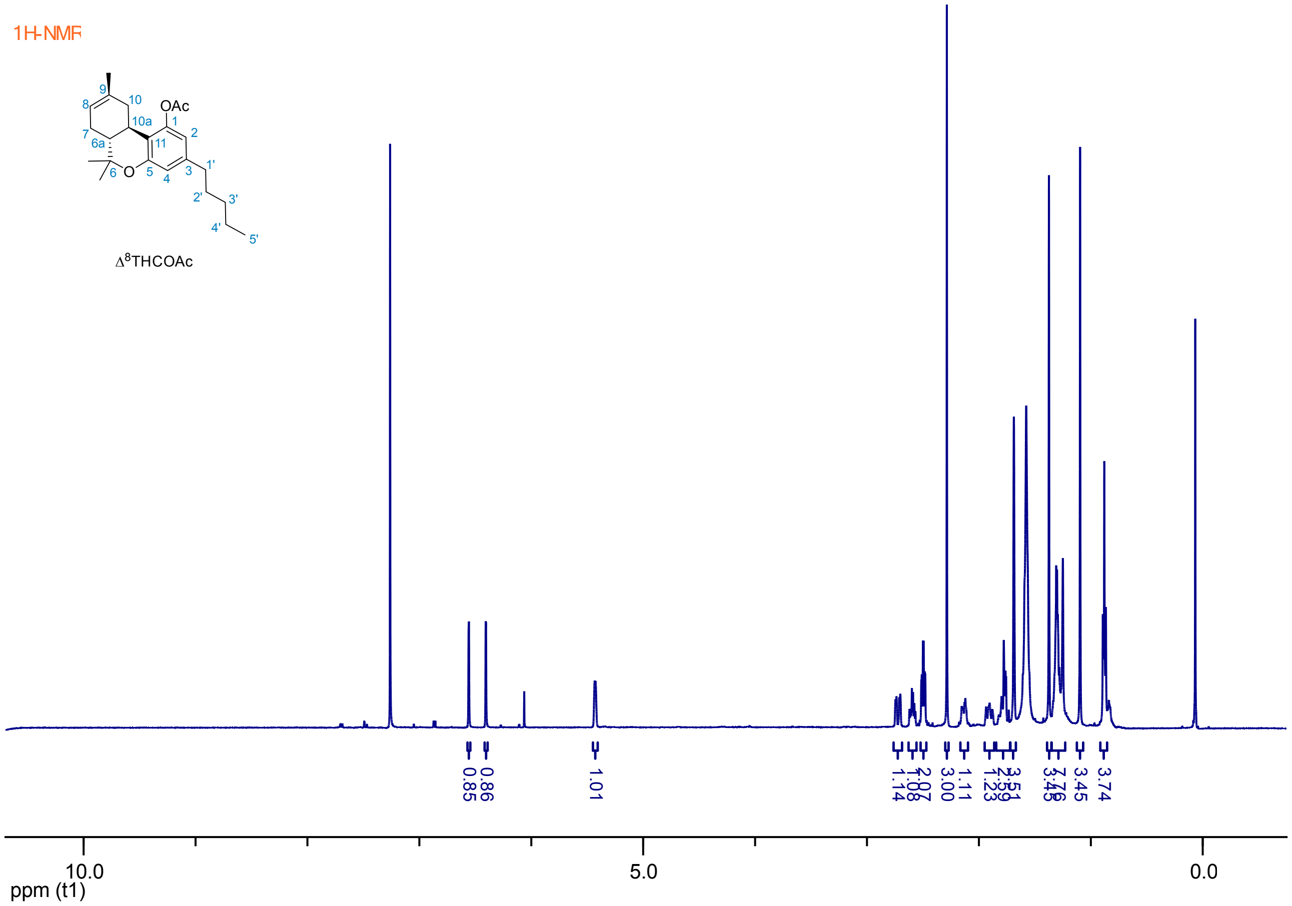
1H-NMR
OAc
Δ8THCOAc
0.85
0.86
1.01
2.07
3.00
1.11
1.23
3.45
3.74
10.0
5.0
0.0
ppm (t1)

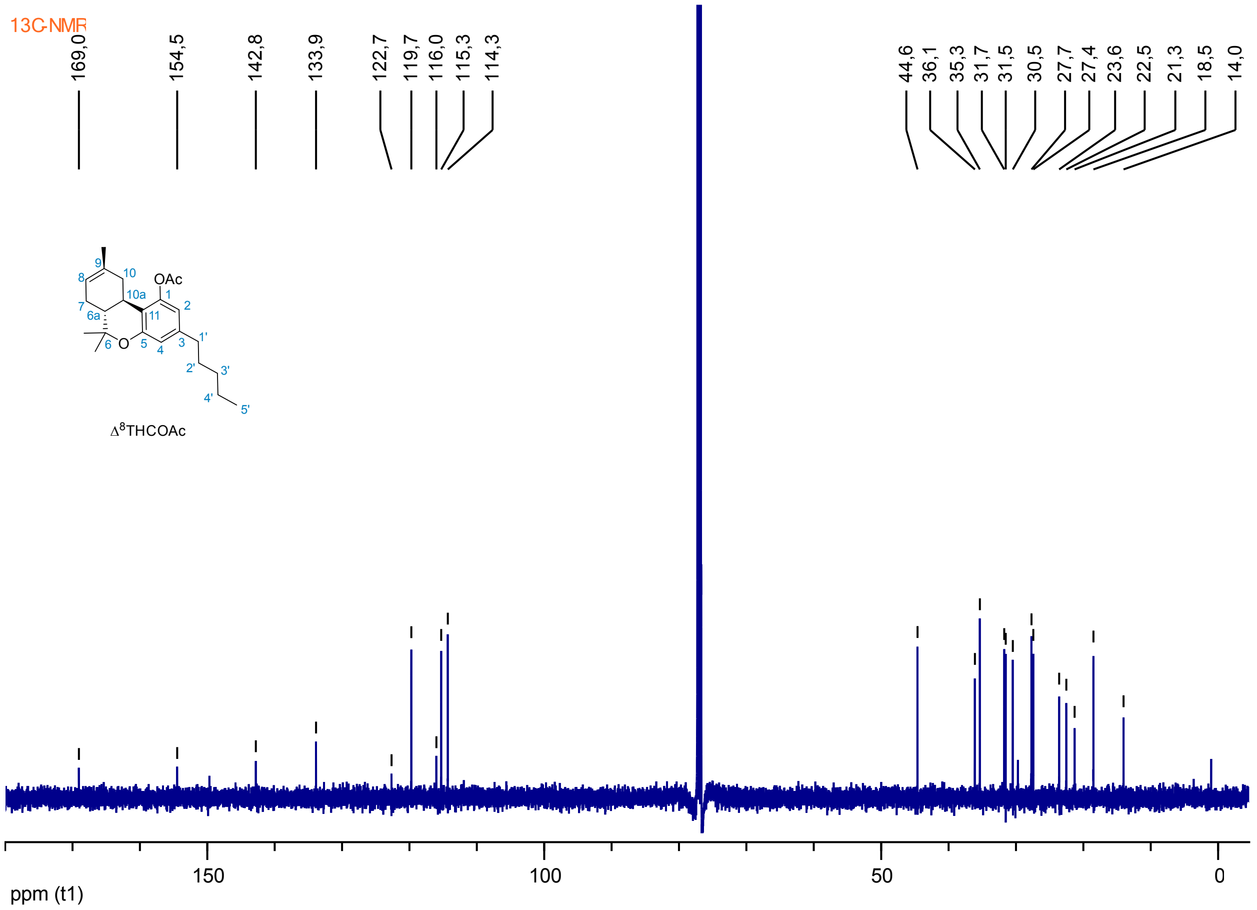
13C-NMR
169,0
154,5
142,8
133,9
122,7
119,7
116,0
115,3
114,3
44,6
36,1
35,3
31,7
31,5
30,5
27,7
27,4
23,6
22,5
21,3
18,5
14,0
OAc
Δ8THCOAc
150
100
50
0
ppm (t1)

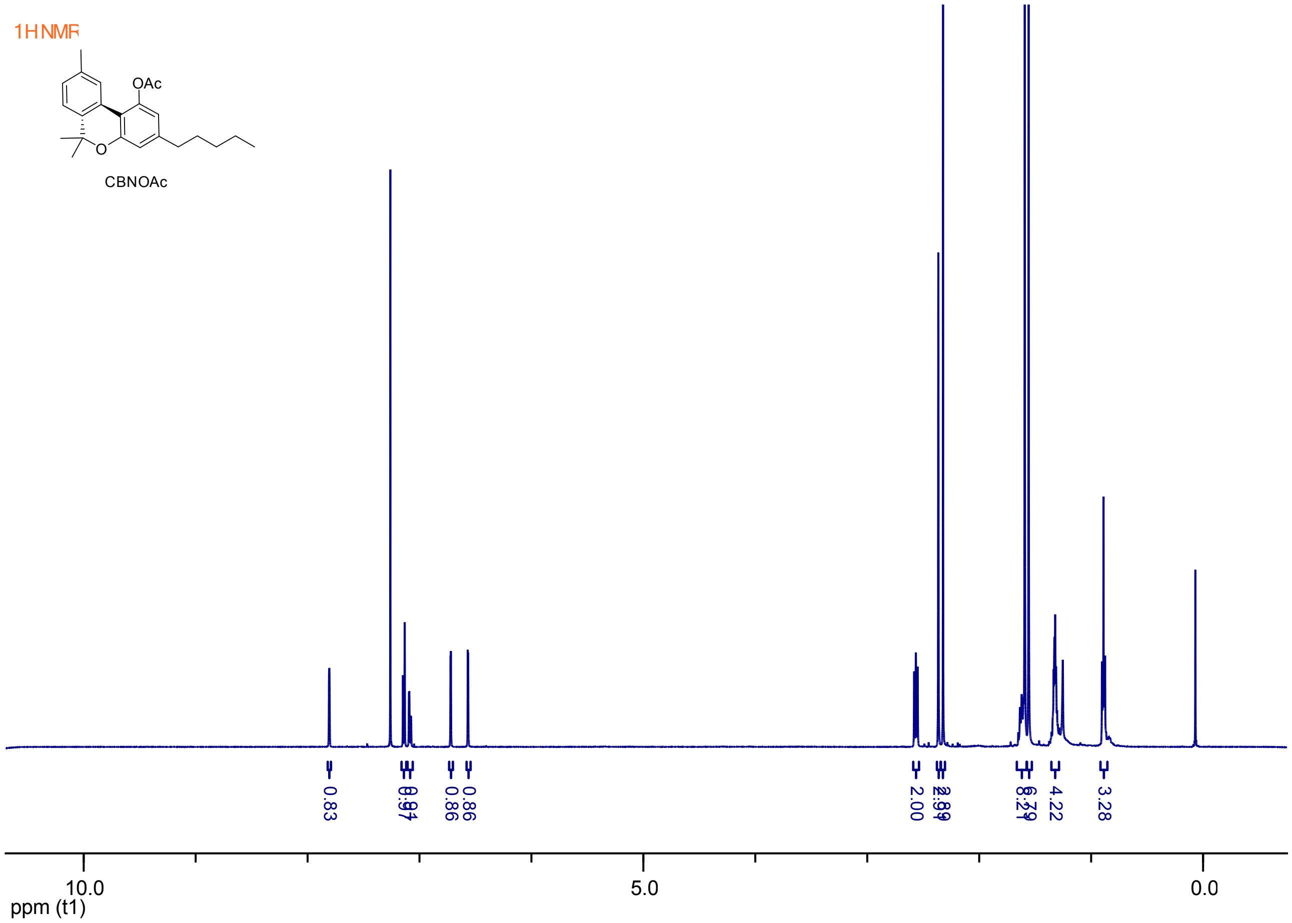

1HNMR
OAc
O
CBNOAc
0.83
0.97
0.91
0.86
0.86
2.00
2.99
6.79
4.22
3.28
10.0
5.0
0.0
ppm (t1)

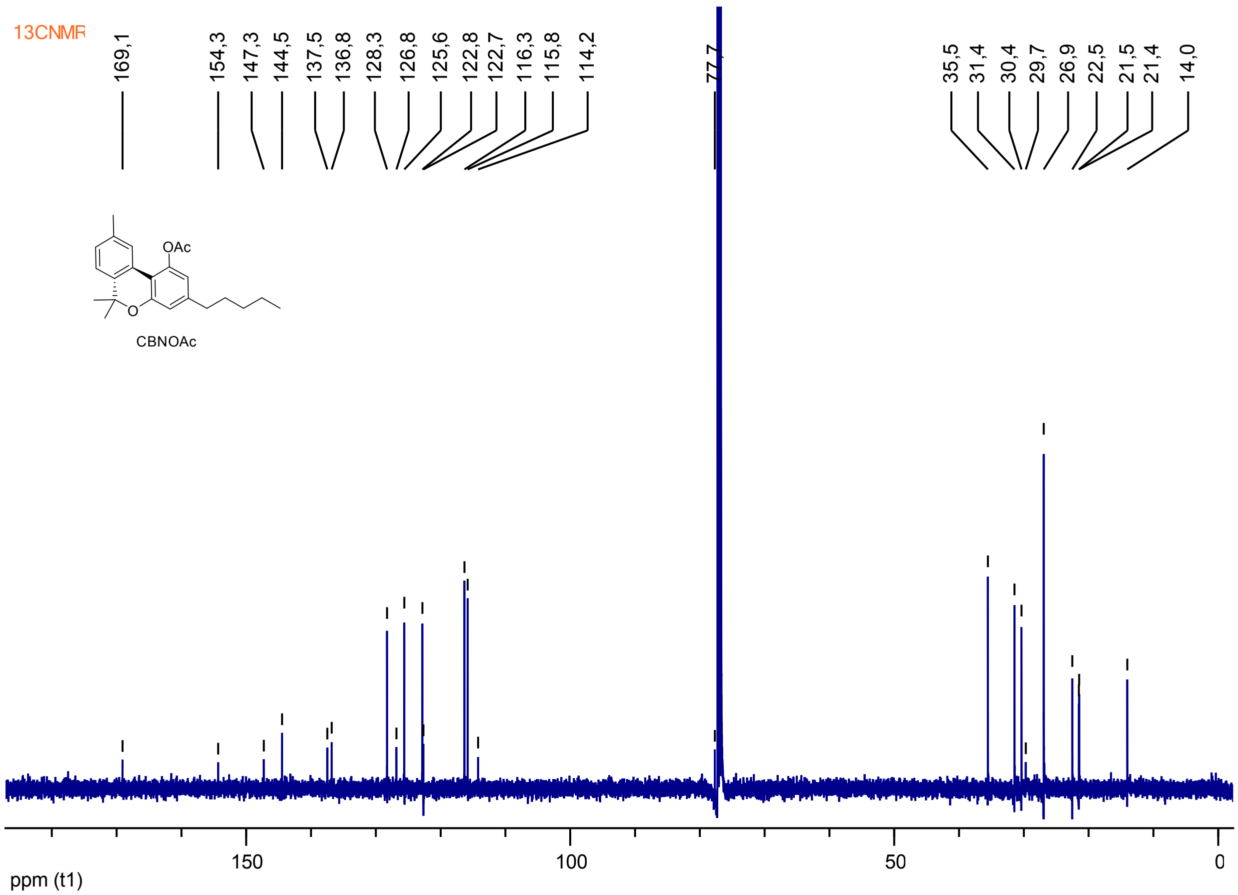

13CNMR
169,1
154,3
147,3
144,5
137,5
136,8
128,3
126,8
125,6
122,8
122,7
116,3
115,8
114,2
77,7
35,5
31,4
30,4
29,7
26,9
22,5
21,5
21,4
14,0
OAc
O
CBNOAc
150
100
50
0
ppm (t1)

A

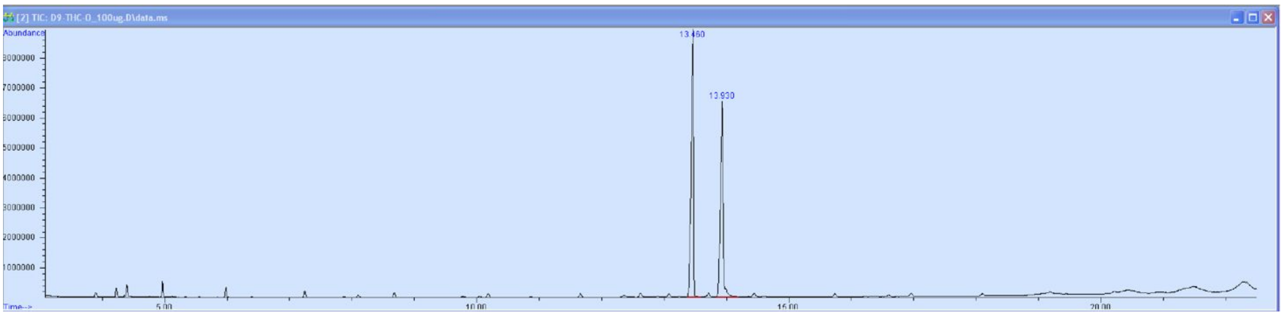
[2] TIC: D9-THC-O_100ug.D\data.ms
Abundance
Time-->

B

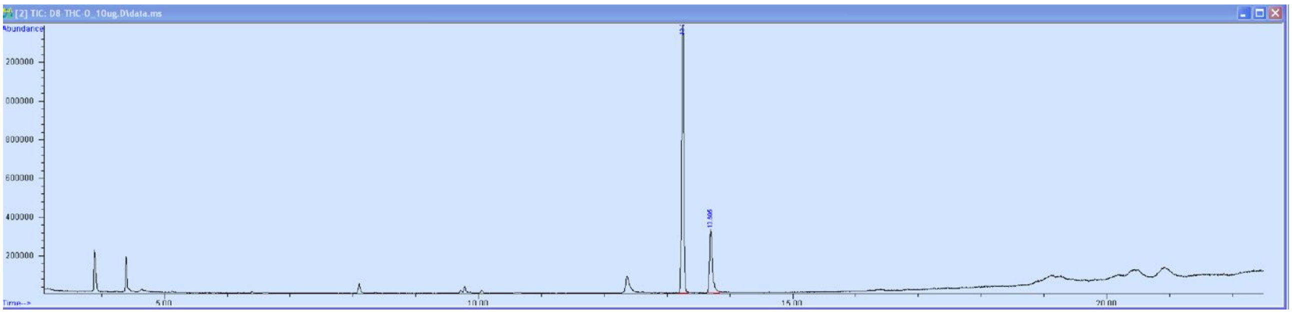
[2] TIC: D8-THC-O_10ug.D\data.ms
Abundance
Time-->

C

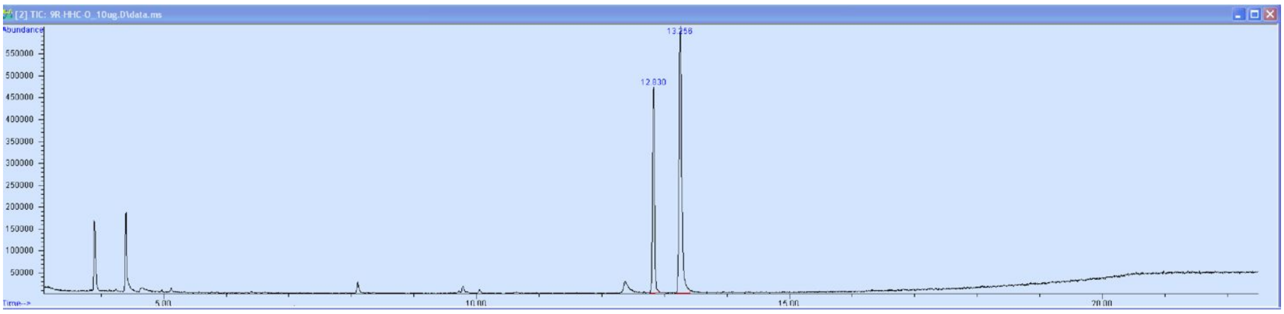
[2] TIC: 9R-HHC-O_10ug.D\data.ms
Abundance
Time-->

D

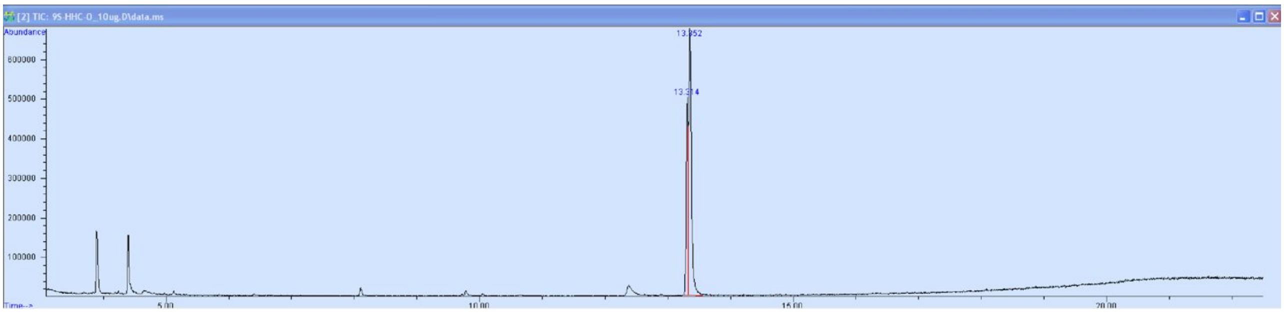
[2] TIC: 9S-HHC-O_10ug.D\data.ms
Abundance
Time-->

E

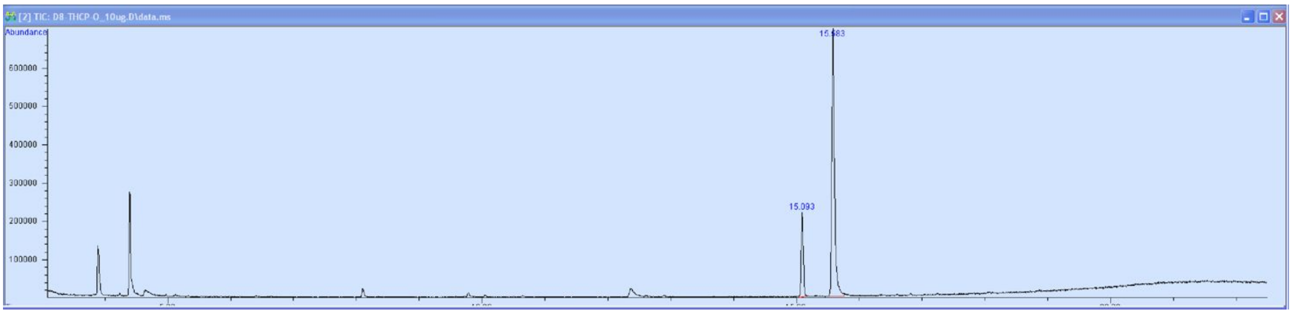
[2] TIC: D8 THCP-O_10ug.D\data.ms
Abundance
600000
500000
400000
300000
200000
100000
15.093

F

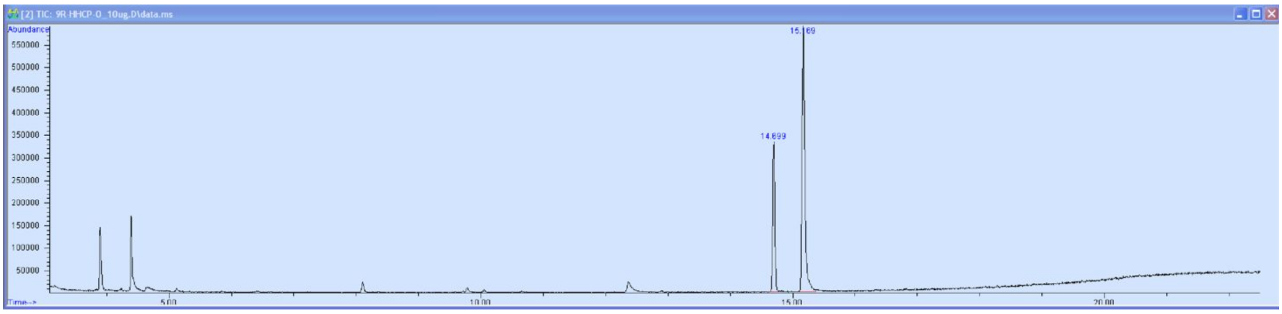
[2] TIC: 9R-HHCP-O_10ug.D\data.ms
Abundance
550000
500000
450000
400000
350000
300000
250000
200000
150000
100000
50000
14.899
Time-->
5.00
10.00
15.00
20.00

G

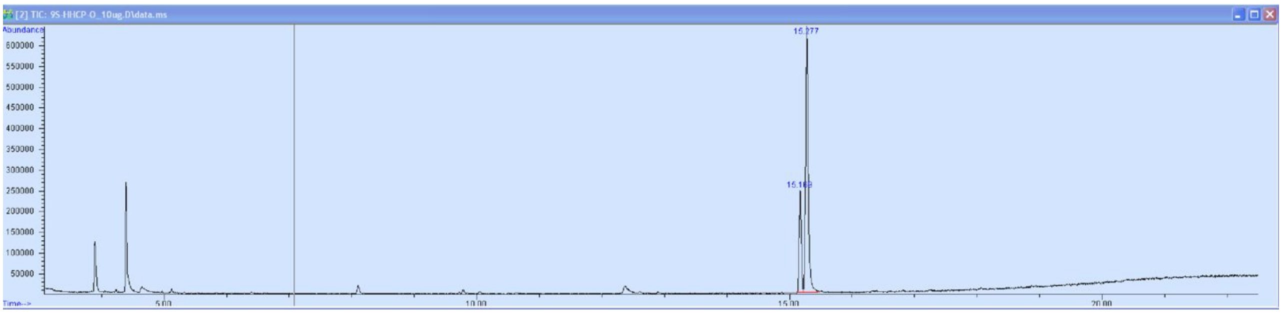
[2] TIC: 9S-HHCP-O_10ug.D\data.ms
Abundance
600000
550000
500000
450000
400000
350000
300000
250000
200000
150000
100000
50000
Time-->
5.00
10.00
15.00
20.00

A

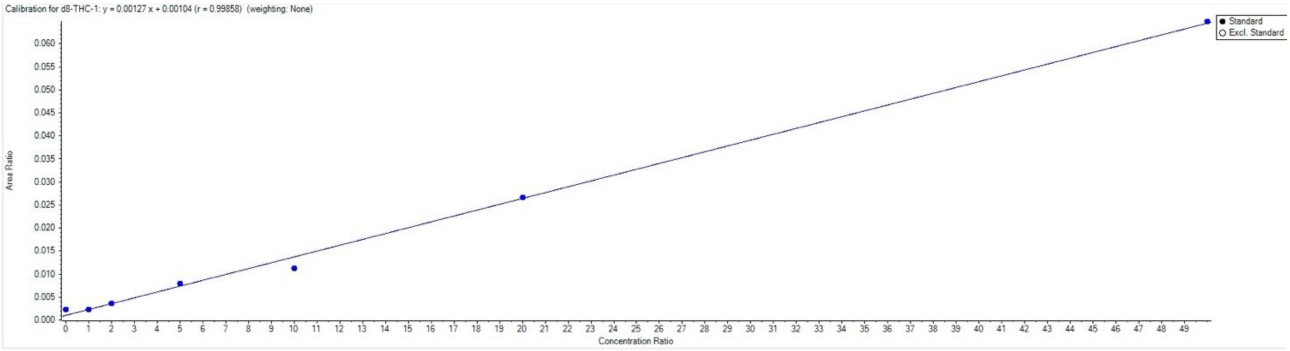
Calibration for d8-THC-1: y = 0.00127 x + 0.00104 (r = 0.99858) (weighting: None)
Standard
Excl. Standard
Area Ratio
Concentration Ratio

B

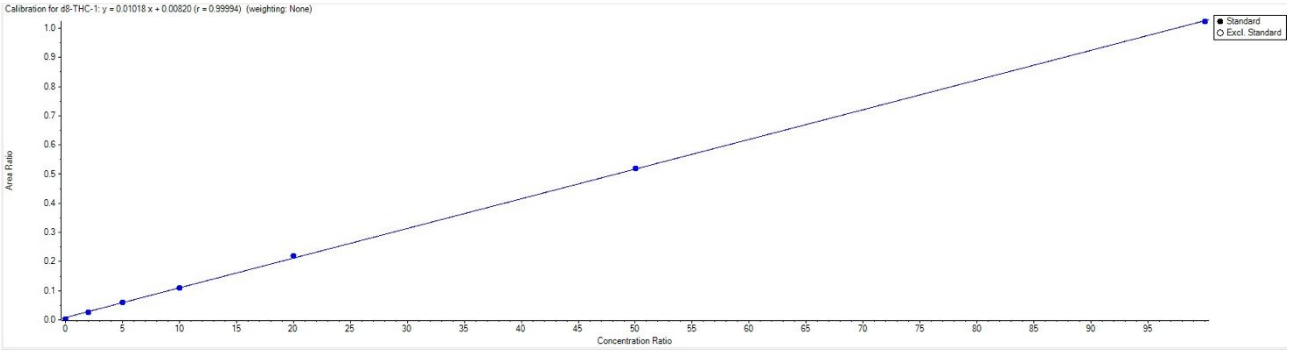
Calibration for d8-THC-1: y = 0.01018 x + 0.00820 (r = 0.99994) (weighting: None)
Standard
Excl. Standard
Area Ratio
Concentration Ratio

C

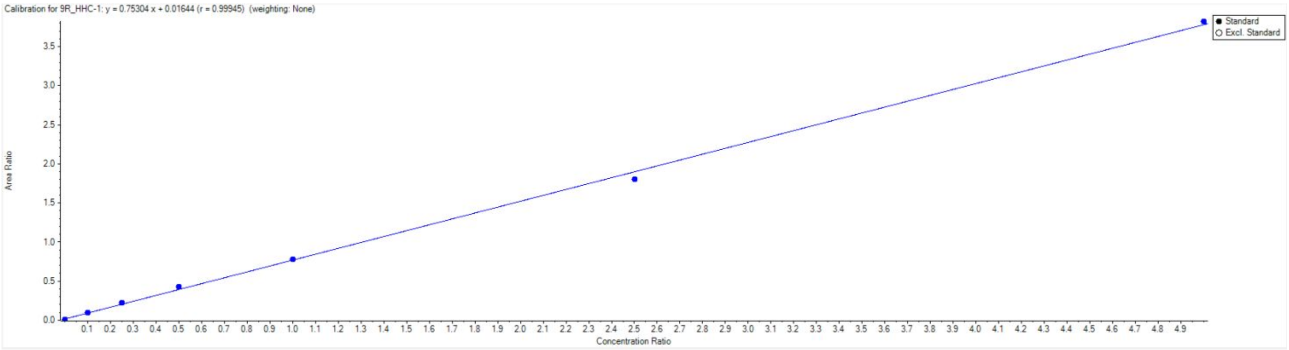
Calibration for 9R_HHC-1: y = 0.75304 x + 0.01644 (r = 0.99945) (weighting: None)
Standard
Excl. Standard
Area Ratio
Concentration Ratio

D

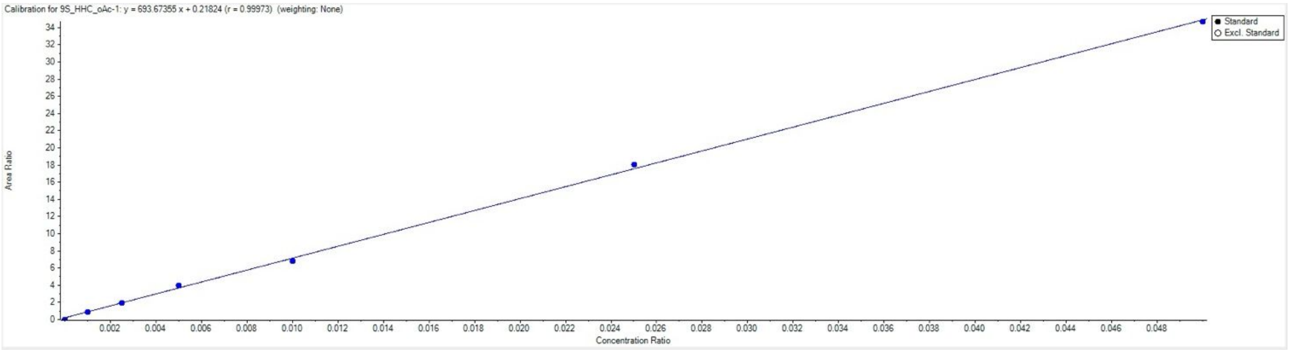
Calibration for 9S_HHC_oAc-1: y = 693.67355 x + 0.21824 (r = 0.99973) (weighting: None)
Standard
Excl. Standard
Area Ratio
Concentration Ratio

E

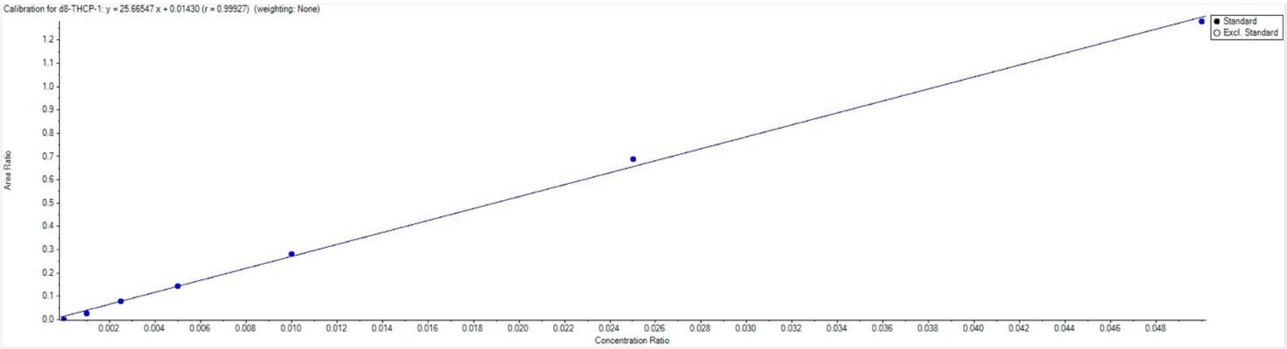
Calibration for d8-THCP-1: y = 25.66547 x + 0.01430 (r = 0.99927) (weighting: None)
Standard
Excl. Standard
Area Ratio
Concentration Ratio

F

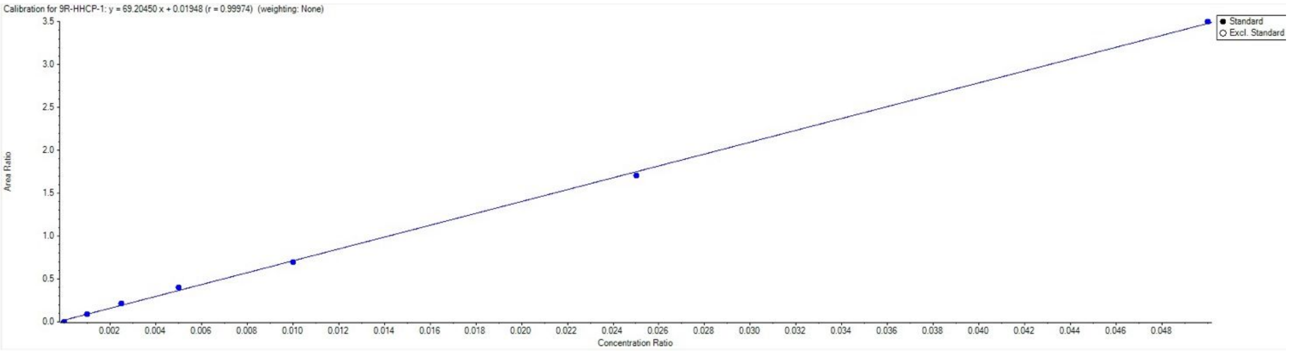
Calibration for 9R-HHCP-1: y = 69.20450 x + 0.01948 (r = 0.99974) (weighting: None)
Standard
Excl. Standard
Area Ratio
Concentration Ratio

G

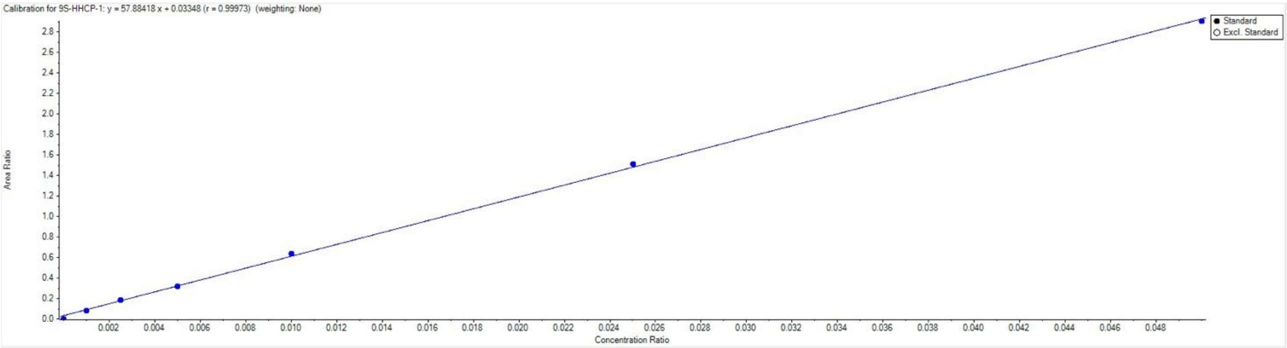
Calibration for 9S-HHCP-1: y = 57.88418 x + 0.03348 (r = 0.99973) (weighting: None)
Standard
Excl. Standard
Area Ratio
Concentration Ratio

A

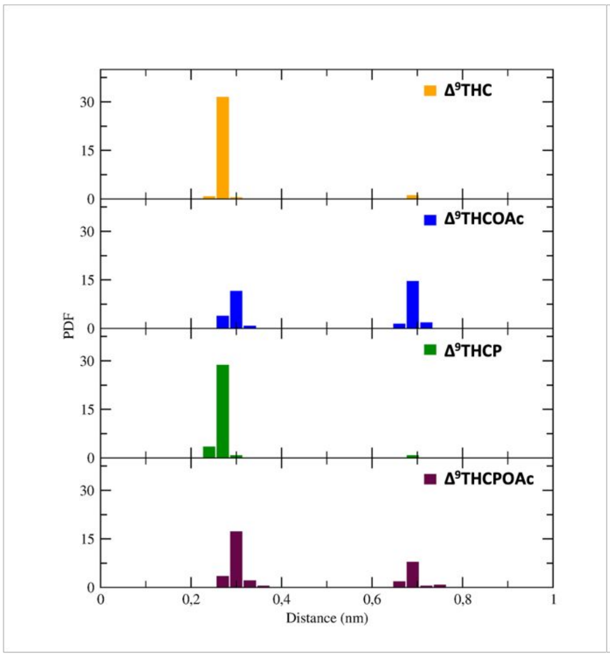
Δ9THC
Δ9THCOAc
Δ9THCP
Δ9THCPOAc
PDF
Distance (nm)
0
15
30
0,2
0,4
0,6
0,8
1

B

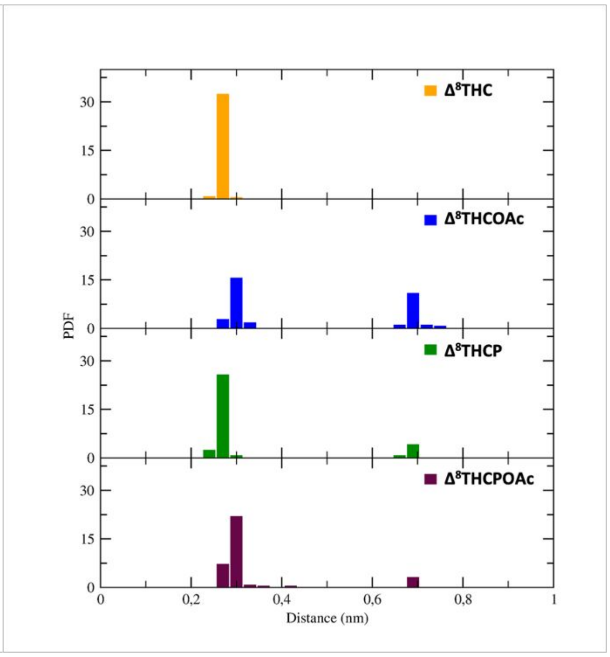
Δ8THC
Δ8THCOAc
Δ8THCP
Δ8THCPOAc
PDF
Distance (nm)
0
15
30
0,2
0,4
0,6
0,8
1

C

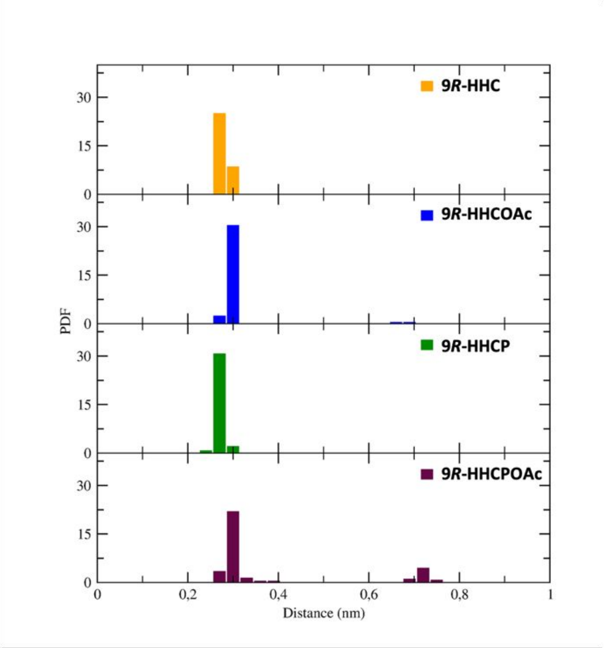
9R-HHC
9R-HHCOAc
9R-HHCP
9R-HHCPOAc
PDF
Distance (nm)
0
15
30
0,2
0,4
0,6
0,8
1

D

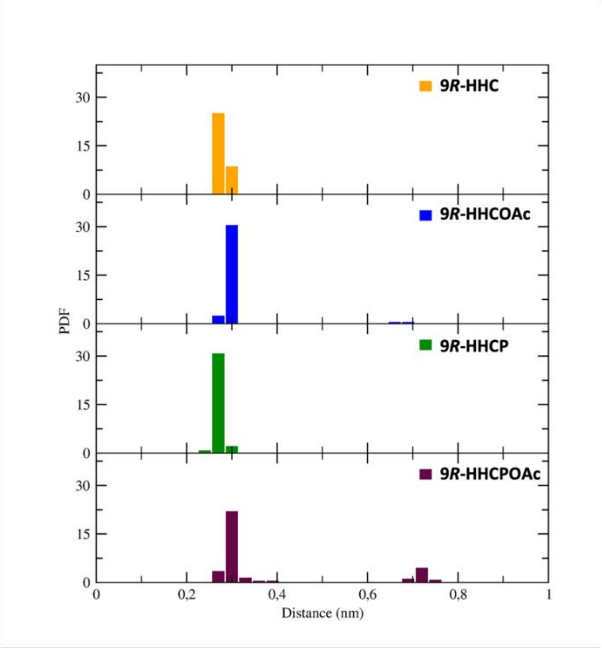
9R-HHC
9R-HHCOAc
9R-HHCP
9R-HHCPOAc
PDF
Distance (nm)
0
15
30
0,2
0,4
0,6
0,8
1

A

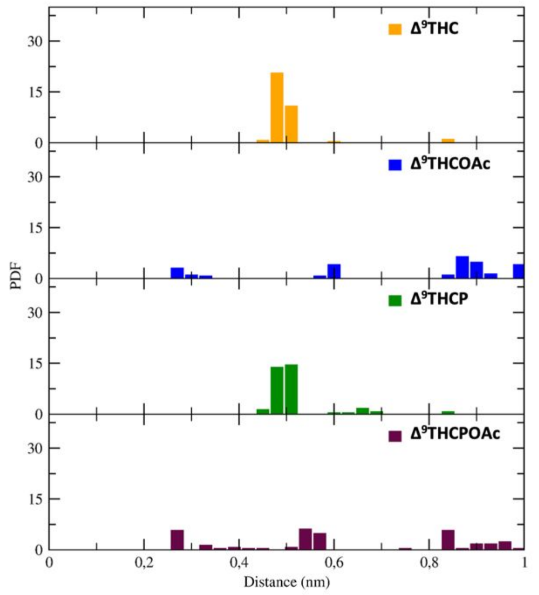
Δ9THC
Δ9THCOAc
Δ9THCP
Δ9THCPOAc
PDF
Distance (nm)

B

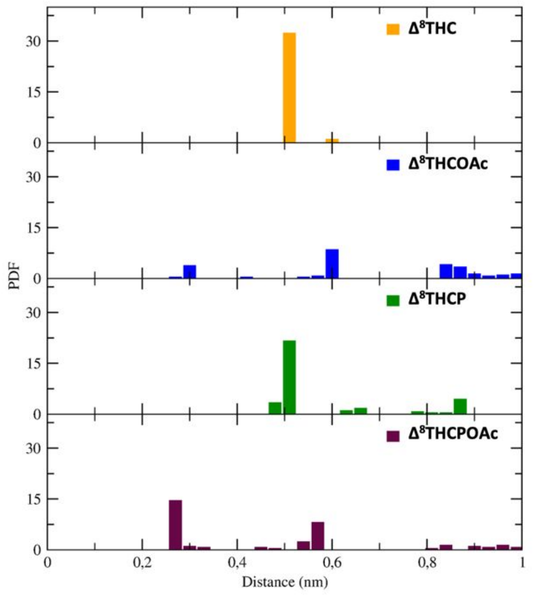
Δ8THC
Δ8THCOAc
Δ8THCP
Δ8THCPOAc
PDF
Distance (nm)

C

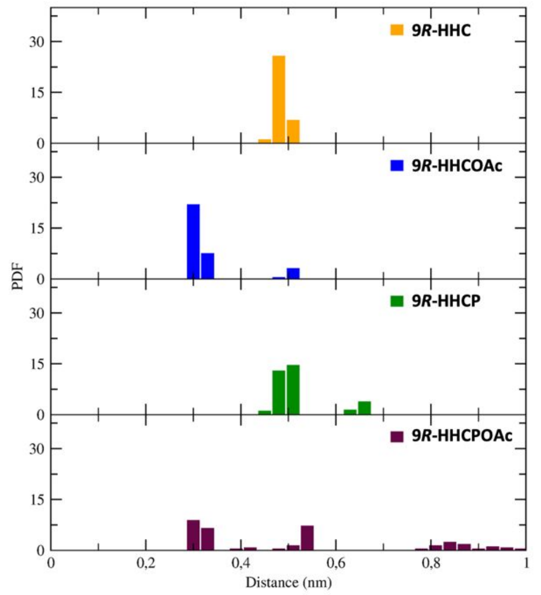
9R-HHC
9R-HHCOAc
9R-HHCP
9R-HHCPOAc
PDF
Distance (nm)

D

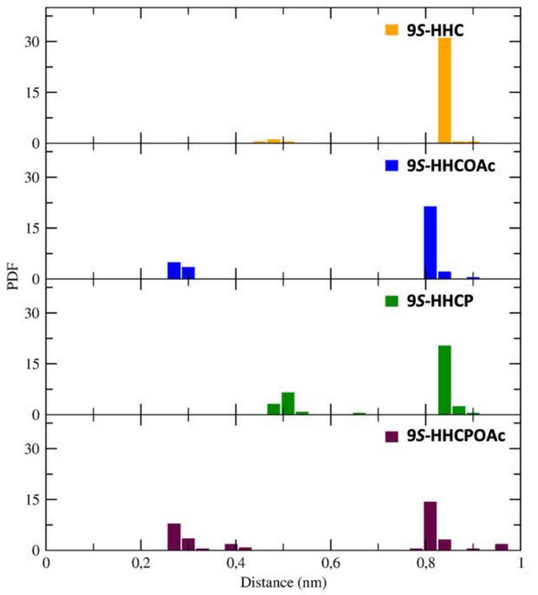
9S-HHC
9S-HHCOAc
9S-HHCP
9S-HHCPOAc
PDF
Distance (nm)

A

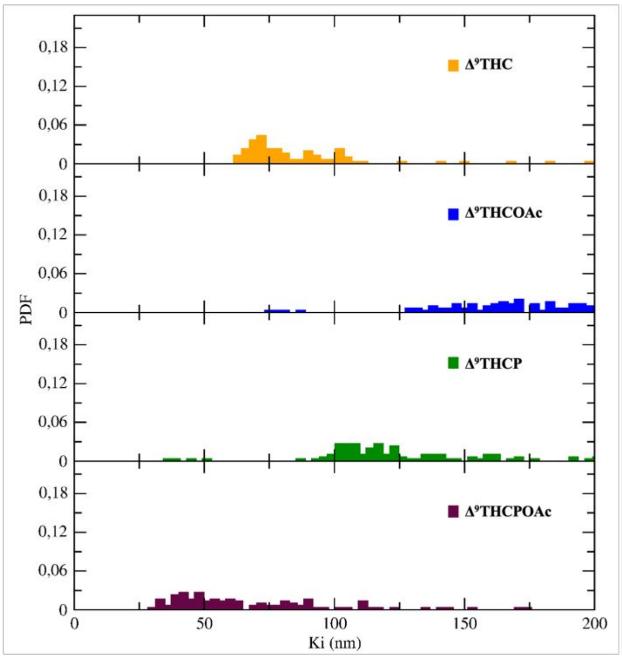
Δ9THC
Δ9THCOAc
Δ9THCP
Δ9THCPOAc
PDF
Ki (nm)

B

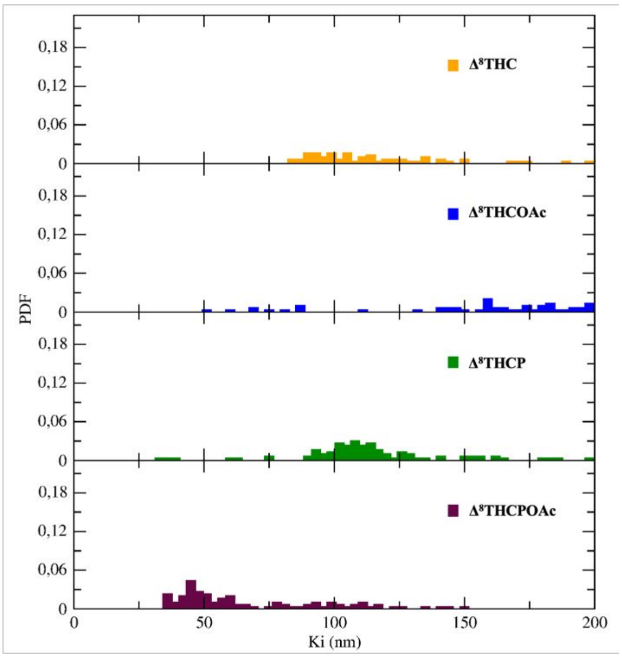
Δ8THC
Δ8THCOAc
Δ8THCP
Δ8THCPOAc
PDF
Ki (nm)

C

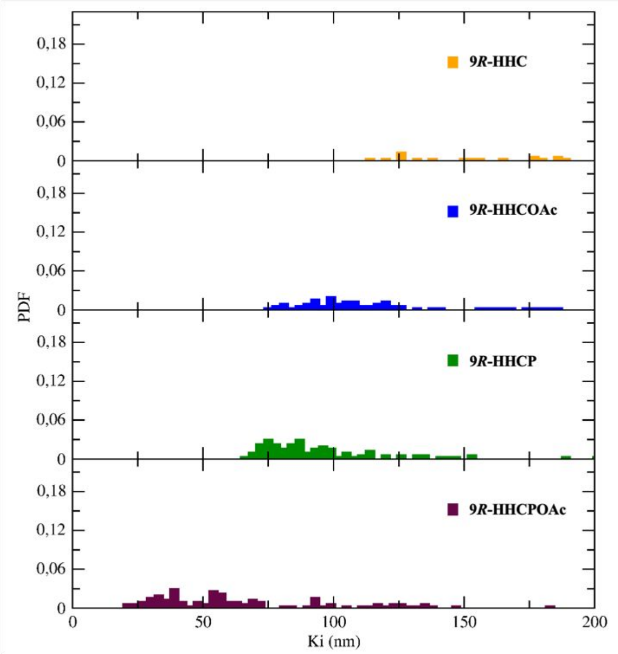
9R-HHC
9R-HHCOAc
9R-HHCP
9R-HHCPOAc
PDF
Ki (nm)

D

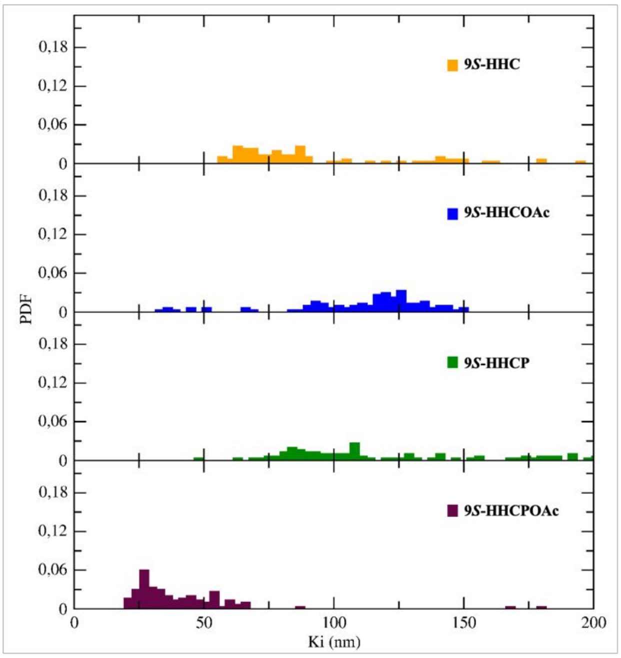
9S-HHC
9S-HHCOAc
9S-HHCP
9S-HHCPOAc
PDF
Ki (nm)
